# Measurement properties of self-assessment instruments for disaster nursing competencies: a systematic literature review

**DOI:** 10.1186/s12912-025-04236-w

**Published:** 2025-12-18

**Authors:** Joachim Beckert, Anita Prasser, Michael Köhler, Michael Ewers

**Affiliations:** Institute of Health and Nursing Science, Charité – Universitätsmedizin Berlin, Corporate Member of Freie Universität Berlin and Humboldt-Universität zu Berlin, Augustenburger Platz 1, 13353 Berlin, Germany

**Keywords:** Disaster nursing, Competencies, Self-assessment, Instrument

## Abstract

**Background:**

The ability of nurses to act competently in disasters is increasingly important, considering the rising disaster events. Self-assessment instruments are used for an evaluation of disaster nursing competencies, yet their scientific rigor has not been systematically evaluated. This review aims to identify self-assessment instruments for disaster nursing competencies based on the International Council of Nurses’ framework and to evaluate their psychometric properties to determine a validated gold standard measurement instrument.

**Methods:**

This review was registered in PROSPERO (CRD42024590462). Studies were eligible for inclusion if they involved nurses, used a self-assessment instrument targeting disaster nursing competencies, and reported psychometric evaluations. Exclusion criteria were studies involving other professionals, lacking an all-hazards approach, psychometrics, or using external assessment instruments. A comprehensive search of seven databases (Cochrane Library, MEDLINE, Embase, CINAHL, PsycINFO, SocINDEX, and ERIC) was conducted up to August 2025 and complemented by reference list screening. Risk of bias assessment, evaluation of measurement properties, and evidence synthesis followed the COSMIN guidelines. A modified GRADE system was used to rate the certainty of the evidence.

**Results:**

Of 758 screened records, eight studies evaluating eight instruments met the inclusion criteria. All instruments revealed conceptual and methodological limitations. Internal consistency was rated sufficient in all studies: quality of evidence “high”; structural validity and reliability were sufficient in seven studies: quality of evidence “very low” to “high”. Content validity: quality of evidence “very low” to “moderate”, construct validity: quality of evidence “high”, and cross-cultural validity: quality of evidence “very low” were sufficiently demonstrated in two, two, and one study, respectively. Criterion validity, measurement error, and responsiveness were not evaluated in any study.

**Conclusions:**

As no instrument met all the requirements for measurement properties, it was not possible to give an unreserved recommendation. Future work should refine existing tools or develop a new, theory-driven instrument aligned with COSMIN, consistently incorporating the ICN framework and ensuring feasibility, transparency, and accessibility.

**Supplementary Information:**

The online version contains supplementary material available at 10.1186/s12912-025-04236-w.

## Background

The number of registered disasters worldwide rose from 90 to 100 per year (1970–2000) to 350–500 (2001–2010). As a result, the competence of nurses to maintain the health and safety of patients and the general population during such events has become increasingly critical [1, 2]. These required competencies fall within the broader scope of disaster nursing, which is defined as “the systematic and flexible utilization of knowledge and skills specific to disaster-related nursing, and the promotion of a wide range of activities to minimize the health hazards and life threatening damage caused by disasters in collaboration with other specialized field“ [3].Nevertheless, nurses, as the largest professional group in healthcare, reported feeling only moderately prepared to apply their knowledge and skills in disaster contexts [4, 5].

To maintain the health and safety of the population during disasters, nurses require specific knowledge and skills, which are encompassed by the concept of disaster nursing competencies. This concept must be distinguished from the related term disaster preparedness, which refers to the state achieved when such competencies are sufficiently developed. The International Council of Nurses (ICN) defines competence as “a level of performance demonstrating the effective application of knowledge, skill and judgment” [6]. This definition underpins the ICN Framework of Core Competencies in Disaster Nursing (hereinafter referred to as the ICN framework), which is regarded as the most established and only evaluated model for describing disaster nursing competencies [6, 7]. Accordingly, in this paper, the term disaster nursing competencies always refers to those defined in the ICN framework. The framework is currently available in a second version from 2019 with two levels of competence [8], which was supplemented by a third competence level in 2023 [9] and is also available in multiple language editions [10, 11]. The ICN framework can be linked to an earlier version, the Disaster Preparedness Framework of the International Coalition for Mass Casualty Education (INCMCE) [12]. The current version of the ICN framework organizes the competencies into eight domains: (1) preparation and planning, (2) communication, (3) incident management systems, (4) safety and security, (5) assessment, (6) intervention, (7) recovery, and (8) law and ethics. To respond effectively to a wide range of disaster scenarios within the framework of an all-hazards approach, nurses require competencies in all eight domains [13].

For an initial assessment of disaster nursing competencies among nurses and to derive corresponding educational needs for workforce development, scientific self-assessment instruments are frequently employed [14]. Self-assessment (as opposed to external assessment) is one of five dimensions employed in scientific approaches to measuring competence, alongside the design approach (theory-driven vs. requirement-oriented), fidelity, and the context of application (individual, institutional, or system level) [15]. Such instruments allow for the systematic collection of subjective perceptions regarding knowledge, skills, and attitudes. They provide valuable insights into competence development processes and can offer long-term support for learning. Due to their practicality and efficiency, they are also widely used in resource-limited settings. However, they are susceptible to bias, such as social desirability and recall bias [16].

To ensure the scientific rigor of such instruments, several standards are available, such as the Standards for Educational and Psychological Testing [17] and the Consensus-based Standards for the selection of health Measurement Instruments (COSMIN standards) [18]. In nursing education research, the COSMIN standards serve as established methodological guidelines for the development and evaluation of measurement instruments [19]. They focus on patient-reported outcome measures, but their methodology can also be applied to other types of measurement instruments. These standards have already been used in several literature reviews [20–23]. They are divided into three core domains: (1) reliability, (2) validity, and (3) responsiveness, and includes a total of ten measurement properties: (1) reliability, (2) internal consistency, (3) measurement error, (4) content validity, (5) criterion validity, (6) structural validity, (7) construct validity, (8) cross-cultural validity (in adaptation studies), (9) measurement invariance (in development studies), and (10) responsiveness, that will be tested in this study. In addition, two further criteria, although not measurement properties in the strict sense, are considered relevant for selecting appropriate instruments: feasibility and interpretability [18].

It remains unclear whether these measurement properties are systematically considered in the development of self-assessment instruments for disaster nursing competencies within nursing education research. Overall, a comprehensive overview of existing instruments and their scientific rigor is still lacking.

This study, therefore, examines the question: What self-assessment instruments currently exist for measuring disaster nursing competencies, and what is their scientific rigor?

The aim is to identify a gold standard self-assessment instrument for measuring disaster nursing competencies to enable its reliable application in research, education, and clinical practice. Based on this, organizations such as hospitals could more effectively monitor the competencies of their staff and, in turn, initiate targeted workforce development measures, thereby sustainably ensuring or even improving the health and safety of staff, patients, and the population in disaster situations.

## Methods

To answer the research questions, a systematic review was chosen as the methodology in accordance with the COSMIN standards [18]. The review was reported following the Preferred Reporting Items for Systematic Reviews and Meta-Analyses (PRISMA) guidelines [24] and was registered in PROSPERO (CRD42024590462). The methodological procedure comprised eight steps: (1) definition of eligibility criteria, (2) development of the search strategy, (3) study selection, (4) data extraction, (5) risk of bias assessment, (6) evaluation and synthesis of measurement properties, (7) assessment of the quality of evidence, and (8) final recommendation.

### Eligibility criteria

The inclusion criteria were: (1) the target population in the studies was nurses; (2) the studies involved the development and evaluation of measurement instruments that claimed to measure disaster nursing competencies; (3) at least one psychometric property of the instrument was examined; and (4) the instruments were self-assessment instruments.

Studies were excluded if they: (1) included nursing assistants, paramedics, or other healthcare professionals; (2) did not use the all-hazards approach; (3) did not examine any psychometric property; or (4) included external rating scales and competency diagnostics (Table S1 in Additional file 1).

### Search strategy

A comprehensive search was conducted in seven international bibliographic databases (Cochrane Library, MEDLINE via PubMed, Embase via Ovid, CINAHL via EBSCOhost, PsycINFO via EBSCOhost, SocINDEX via EBSCOhost, and ERIC via EBSCOhost) from their inception to August 2025 without language restrictions. The reference lists of included articles were hand-searched to ensure that all relevant studies were included.

The search syntax included the following basic keywords and Boolean operators: “disaster”, “disaster nursing”, “competency”, “professional competence”, “skill”, “knowledge”, “ability”, “capacity”, “capability”, “judgement”, “attitude”, “preparedness”, “nurses”, “index”, “instrument”, “measure”, “questionnaire”, “profile”, “scale”, “score”, “status”, “survey” and “tool”. The search syntax also included synonyms and the most relevant technical terms of the basic keywords in each concept. Where appropriate, a shortened term was used with the wildcard character to maximize sensitivity while striving for appropriate precision (Table S2 in Additional file 1). The search syntax was adapted to the respective database (Table S3 in Additional file 1).

In addition, both the COSMIN search filter for measurement properties [25] and the patient-reported outcome measures (PROMs) search filter [26] were applied, in accordance with COSMIN standards, in a version adapted for this systematic literature review. Both filters were adapted for each database (Table S4-S5 in Additional file 1).

### Selection process

Based on the predefined inclusion and exclusion criteria, the review team developed a screening protocol to guide the study selection process. On this basis, calibration was performed to review and refine the instructions, and training was provided for inexperienced team members. Search results were imported into the web-based tool Systematic Review Accelerator [27] and deduplicated using the Systematic Review Deduplicator [27].

**Fig. 1:**
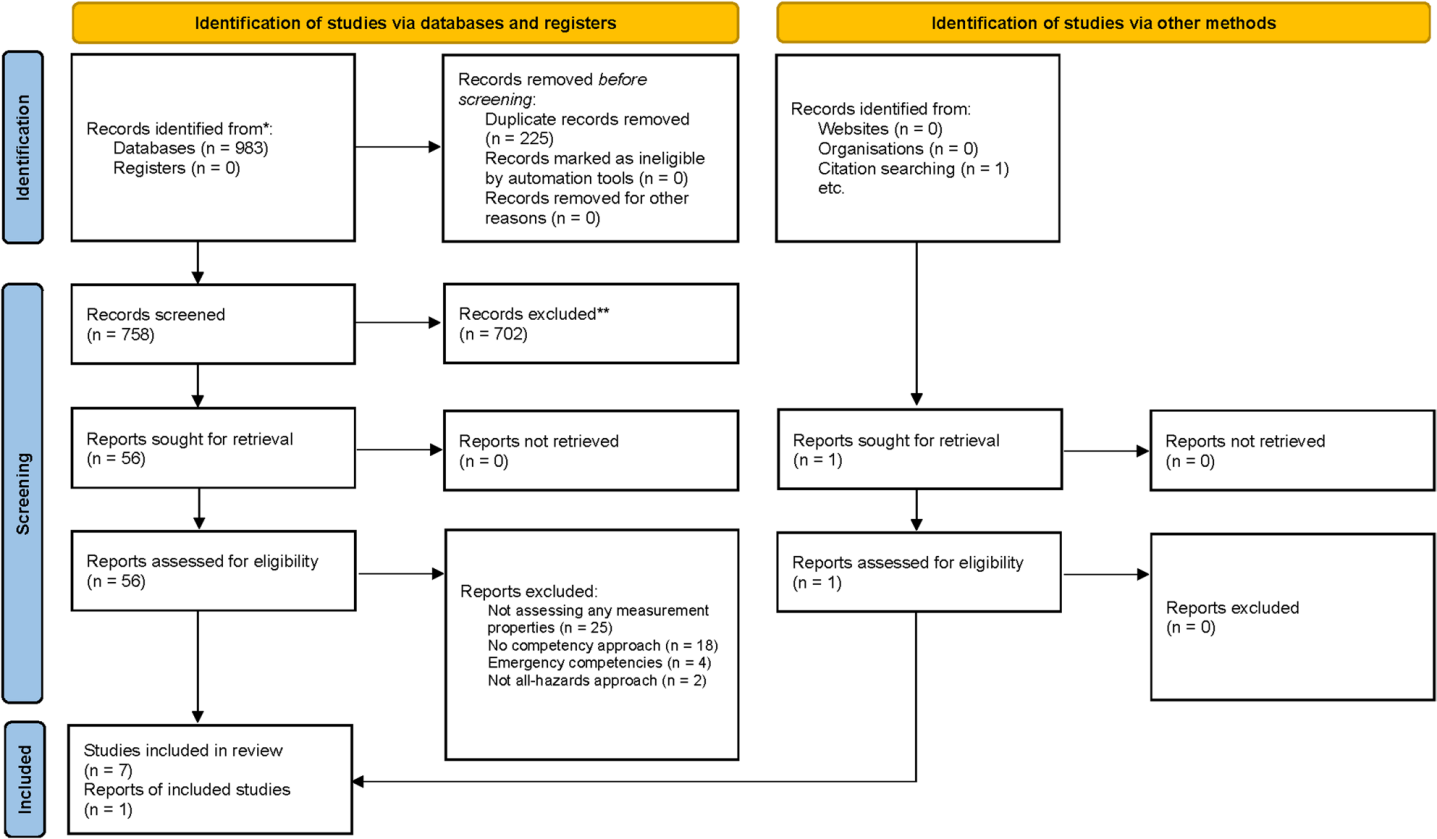
PRISMA flow diagram

Two members of the review team (JB and AP) independently screened titles/abstracts and full texts according to the protocol. Discrepancies during full-text screening were resolved by a third team member (MK). Reasons for exclusion are documented in the PRISMA flow diagram (Fig. 1). The studies included through this process formed the basis for subsequent data extraction and evaluation of psychometric quality.

### Data extraction

Data extraction was also conducted independently by JB and AP using the COSMIN Excel template (Additional file 2). Before extraction, another calibration round was performed, and any disagreements were resolved by MK. The following data were extracted: (1) general information for identifying the instrument, (2) information on the development process, and (3) psychometric properties.

General information included: (a) instrument name, (b) language, (c) country of development, (d) construct of interest, (e) origin of the construct, (f) target population, (g) sample size, (h) intended context of use, (i) number of items, (j) names and number of subscales, (k) response options, and (l) number of adaptations.

Information on instrument development was extracted from the respective studies. The psychometric properties extracted included: (a) content validity, (b) structural validity, (c) internal consistency, (d) cross-cultural validity/measurement invariance, (e) reliability, (f) measurement error, (g) criterion validity, (h) construct validity, and (i) responsiveness.

### Risk of bias assessment

The quality of the included studies was assessed by two members of the review team (JB and AP) based on ten COSMIN standards: (1) instrument development, (2) content validity, (3) structural validity, (4) internal consistency, (5) cross-cultural validity (in adaptation studies) / measurement invariance (in development studies), (6) reliability, (7) measurement error, (8) criterion validity, (9) construct validity, and (10) responsiveness. Each standard was rated on a four-point scale: “very good,” “adequate,” “doubtful,” or “inadequate”. According to COSMIN, the lowest rating within each standard was used to determine the overall methodological quality for that standard. Discrepancies were resolved through consensus with a third reviewer (MK).

### Quality assessment of psychometric properties and synthesis

In addition to evaluating the methodological quality of the studies, the reported psychometric properties of the instruments themselves were independently assessed by two members of the review team (JB and AP). Discrepancies were resolved through discussion with the third reviewer (MK). Content validity was assessed based on ten COSMIN criteria (Additional file 2), considering the full body of available evidence, including both the original development studies and the review team’s own evaluation. The results of both assessments were synthesized qualitatively. The overall rating for content validity could be classified as sufficient (+), insufficient (–), inconsistent (±), or indeterminate (?).

The remaining psychometric properties, structural validity, internal consistency, cross-cultural validity/measurement invariance, reliability, measurement error, criterion validity, construct validity, and responsiveness, were assessed according to the COSMIN criteria for good measurement properties. Each property was rated as sufficient (+), insufficient (–), or indeterminate (?).

### Grading the quality of evidence

Two members of the review team (JB and AP) independently assessed the quality of evidence using the modified GRADE approach (Grading of Recommendations, Assessment, Development and Evaluation). This method allows for a structured and comparable appraisal of evidence quality. Each psychometric property was initially rated as “high”. The rating could then be downgraded based on four criteria: risk of bias, inconsistency, indirectness, and imprecision. The final quality levels were classified as “high,” “moderate,” “low,” or “very low”.

The criterion of imprecision was not applied to content validity, structural validity, and cross-cultural validity, as the COSMIN risk of bias checklist already defines minimum sample size requirements for these properties. If the overall rating of a measurement property was indeterminate, labeled as question mark (?), the quality of the instrument could not be assessed, and consequently, the quality of evidence for that property could not be rated.

### Recommendation for use of instruments

Instruments are recommended for use if high-quality evidence demonstrates that all relevant measurement properties are sufficient. Conversely, if high-quality evidence indicates that one or more properties are insufficient, the instrument is not recommended. In all other cases, a final recommendation cannot be made at this time, as further studies are needed to evaluate the measurement properties [18].

## Results

The following section first outlines the study selection process to ensure transparency in how the evidence base was identified and to allow readers to evaluate the comprehensiveness and rigor of the inclusion procedure. This is followed by a description of the characteristics of the included studies, as these details are essential for contextualizing the findings and assessing their applicability. Subsequently, for each COSMIN standard, the Rating of Results and the Quality of Evidence are reported to provide a systematic, standardized, and comparable assessment of the psychometric properties that could be evaluated.

### Study selection

The search strategy identified 983 records across seven databases. After removal of duplicates, 758 studies were screened based on the eligibility criteria, and 56 full texts were assessed for inclusion. One additional study was identified through the reference list screening of included studies. Studies were excluded during screening and assessment in accordance with the eligibility criteria. In total, eight studies involving eight measurement instruments met the inclusion criteria and were included in the review (Fig. 1).

### Study characteristics

Of the eight included studies, six were development studies [28–33] and two were adaptation studies [34, 35], all reporting on the development or adaptation of instruments to assess disaster nursing competencies between 2016 and 2024 (Table 1).

**Table 1:** Characteristics of the instruments used to measure disaster nursing competencies

Instrument Name (abbr.) (ref)	Country/ Language	Construct of Interest	Origin of the Construct/Adaptation	Study Participants/ Setting	Number of Items	Subscales (Number of Items)	Response Options	Administration	Reported measurement properties
Nurses‘ Disaster Response Competencies Assessment Questionnaire (NDRCAQ) [28]	Brazil/ Portugese	Nurse Disaster Response Competencies	ICN Framework	Practicing nurses	41	Care of the Community (14) Care of the Individual and Family (15) Psychological Support and Care of Vulnerable Populations (12)	5-point Likert scale	Self-assessment	Content Validity Structural Validity Internal Consistency Reliability
Disaster Nursing Competency Scale (DNCS) [29]	Iran/ Not reported	Disaster Nursing Competencies	ICN Framework	Nurses	50	Management Competency (12) Ethical and Legal Competency (6) Personal Competency (9) Technical Competency (23)	5-point Likert scale	Self-assessment	Content Validity Structural Validity Internal Consistency Reliability
Disaster Nursing Core Competencies Scale (DNCCS) [30]	Saudi Arabia/ Not reported	Core Competencies of Disaster Nursing	ICN Framework and additional literature	Emergency Nurses in hospital setting	29 (only Core competencies subscale)	Core Competencies of Disaster Nursing (29)	10-point Likert scale	Self-assessment	Content Validity Internal Consistency Reliability
Instrument Nurses‘ Core Competencies for Public Health Emergencies (NCC-PHE) [31]	China/ Not reported	Core Competencies of Disaster Nursing	ICN Framework	Nurses	47	Prevention Competencies (9) Preparation Competencies (7) Response Competencies - Basic Level (17) Response Competencies - Advanced Level (9) Recovery Competencies (5)	5-point Likert scale	Self-assessment	Content Validity Structural Validity Internal Consistency Reliability Construct Validity
Nurse Competency Assessment Scale in Disaster Management (NCASDM) [32]	Turkey/ Not reported	Competencies related to disaster management	ICN Framework	Nurses	43	Disaster Preparedness (9) Disaster Response (34)	5-point Likert scale	Self-assessment	Content Validity Structural Validity Internal Consistency Reliability
Disaster Nursing Preparedness – Response Competency Scale (DNPRCS) [33]	South Korea/ Not reported	Disaster Nursing Preparedness and Response Competency	ICN Framework	Nurses	34	Communication and Information Sharing (7) Education (3) Ethical and Legal Practice (6) Psychological Care and Vulnerable Population Care (6) Individual and Family Care (5) Cooperative Care (4) Safety Care (3)	5-point Likert scale	Self-assessment	Content Validity Structural Validity Internal Consistency Construct Validity
Nurses’ Perceptions of Disaster Core Competencies Scale (NPDCCS) [34]	China/ Nor reported	Disaster Nursing Core Competencies	International Coalition for Mass Casualty Education (INCMCE) Disaster preparedness framework / Adaptation of Celik [36]	Nurses	45	Critical Thinking Skills (4) General Diagnostic Skills (13) Special Diagnostic Skills (6) Technical Skills (14) Communication Skills (8)	5-point Likert scale	Self-assessment	Content Validity Structural Validity Internal Consistency Reliability
Competencies for Disaster Nursing Management Questionnaire (CDNMQ) [35]	Turkey/ Not reported	Competencies for Disaster Nursing Management	ICN Framework and additional literature / Adaptation of the DNCCS	Emergency nurses in hospital setting	29 (only core competencies subscale)	Core Competencies of Disaster Nursing (29)	5-point Likert scale	Self-assessment	Content Validity Structural Validity Internal Consistency Reliability

All instruments were self-assessment tools comprising between 34 [33] and 50 [29] items. Most instruments used 5-point Likert scales, except Al Thobaity, Williams [30], who employed a 10-point scale. The target population in most studies consisted of registered nurses [28, 29, 31–34], while two studies specifically targeted emergency nurses in hospital settings [30, 35].

None of the studies included in the review assessed all relevant measurement properties (Table 3). Across studies, content validity and internal consistency were evaluated in all cases, while structural validity, reliability, cross-cultural validity, and construct validity were examined only occasionally. Measurement invariance, measurement error, criterion validity, and responsiveness were not assessed in any study.

All instruments were requested from the respective authors; however, none were made available. Consequently, only six instruments could be fully analyzed and evaluated, as only these had their items published in the respective articles. No information was available regarding feasibility aspects such as cost, licensing, or time required for administration. Data on interpretability were likewise unavailable.

### Instrument development: rating of results and quality of evidence

The development of measurement instruments is critical to their subsequent scientific quality. Development can follow either a theory-driven or a requirements-based approach. All instruments included in this review were, in a broad sense, grounded in the ICN framework for Disaster Nursing Competencies, regardless of the specific version used, as their theoretical foundation. Four building on the first edition from 2009 [28, 30, 33, 34], three on the revised 2019 edition [29, 31, 32], and one on its predecessor, the Disaster Preparedness Framework of the ICMCE [34].

Upon closer inspection, only one instrument, the NDRCAQ, is structured according to the eight dimensions of the ICN framework [28]. However, it only covers dimensions related to the “Preparedness” and “Recovery” phases of disaster management. The remaining multidimensional instruments, which comprise between two [32] and seven [33] subscales, are structured either according to the phases of the disaster management cycle [31, 32] or thematically [29, 33, 34]. In two studies, disaster nursing competencies are measured using a single subscale [30, 35], while additional subscales assess constructs such as nurses’ roles or barriers to the further development of disaster nursing [30]. While the NCC-PHE also covers level 2 of the ICN frameworks’ competencies (advanced practice), all other instruments are confined to level 1, which is relevant for all licensed nurses.

Four studies employed qualitative methods to identify appropriate items for instrument development [29, 31–33]. Reports on the use of interview guides, group meetings with trained moderators, audio recordings, or transcripts were rare. The DNCS and DNPRCS studies collected qualitative interview data until saturation was achieved.

Pilot testing was conducted in most cases (*n* = 7), though never as a separate study. Nevertheless, the pilot samples generally reflected the target population. In five studies, nurses were asked to assess item comprehensibility, though it was not always clear whether the final item versions were used. Cognitive interviews were commonly applied, although not consistently documented. Sample sizes typically adhered to recommended thresholds: 4–6 participants for qualitative and ≥ 30 for quantitative testing. Rarely was it reported whether interviews were recorded or transcribed, whether items were revised afterward, or how many developers were involved in the process.

Only the development process of the DNCS met all methodological standards. However, no dedicated pilot study was conducted to assess item comprehensibility in that case (Table 2).

**Table 2:** Rating of instrument development

Instrument	Concept elicitation		Pilot study
	QS		QS
NDRCAQ	D		n. a.
DNCS	VG		n. a.
DNCCS	D		D
NCC-PHE	D		D
NCASDM	A		D
DNPRCS	D		D
NPDCCS	D		D
CDNMQ	D		D

Note. A = Adequate; D = Doubtful; I = Inadequate; n. a. = Not applicable; QS = Quality of Study; VG = Very good

### Content validity: rating of results and quality of evidence

The overall assessment of content validity was inconsistent in most studies (*n* = 5). Only the studies on the DNCS and the CDNMQ were rated as having sufficient content validity (Table 3).

Regarding item relevance, comprehensiveness, and comprehensibility, the findings were mixed (Table S6 in Additional file 1). In terms of relevance, six instruments received inconsistent ratings. While the original development studies judged the items to be relevant, the review team found that more than 15% of the items were not relevant to the construct of disaster nursing competencies. Examples include: psychological or preventive measures in the NDRCAQ; identification of vulnerable groups in the DNCCS; personality traits, context-specific awareness, and non-nursing or highly specialized skills in the NCC-PHE; logistics and resource management in the NCASDM; macro-level inter-organizational collaboration, self-reflection, and community knowledge dissemination in the DNPRCS; and vulnerability analysis, psychosocial competencies, and specific clinical tasks in the NPDCCS. These inconsistencies align with the overall doubtful quality of the studies regarding item relevance (*n* = 6).

Comprehensiveness was judged to be sufficient only for the DNCS and NPDCCS. All remaining instruments lacked items representing key concepts of the disaster nursing competencies construct. For example, the NDRCAQ lacked items on communication competencies; the DNCCS and NCC-PHE lacked items related to safety and security; the NCASDM lacked items on recovery; and the DNPRCS lacked items on assessment procedures. The methodological quality of the studies concerning comprehensiveness was likewise often rated as doubtful.

Item comprehensibility was rated as sufficient in most instruments (*n* = 5), although study quality in this regard was mostly doubtful. In the cases of the NDRCAQ, DNCCS, and NCC-PHE, no nurses were consulted on item clarity, resulting in an indeterminate rating.

**Table 3:** Content validity: rating of measurement properties, study quality, and quality of evidence

			NDRCAQ	DNCS	DNCCS	NCC-PHE	NCASDM	DNPRCS	NPDCCS	CDNMQ
Content Validity	Relevance	R	±	+	±	±	±	±	±	+
		QS	D	D	D	D	D	A	A	D
	Comprehensiveness	R	–	+	–	±	±	?	+	?
		QS	D	D	D	D	D	A	D	D
	Comprehensibility	R	?	+	?	?	+	+	+	+
		QS	D	D	D	D	D	D	D	D
	Overall	R	±	+	±	±	±	±	±	+
		QE	Very low	Moderate	Very low	Low	Moderate	Low	Very low	Very low

Note. A = Adequate; D = Doubtful; I = Inadequate; QE = Quality of Evidence; QS = Quality of Study; R = Rating; VG = Very good; + = Sufficient; – = Insufficient; ? = Indeterminate; •± = Inconsistent

### Strucutral validity: rating of results and quality of evidence

Structural validity was assessed using either exploratory (*n* = 5) or confirmatory (*n* = 5) factor analyses. All reported fit indices fell within acceptable ranges (Table S7 in Additional file 1), resulting in a sufficient rating for structural validity in most cases (*n* = 7) (Table 4). In the DNCS study, it remained unclear whether a principal axis factor analysis or a principal component analysis was conducted.

The methodological quality of studies was rated as “very good” whenever a confirmatory factor analysis was used (*n* = 5). In contrast, studies employing exploratory factor analyses were rated as having doubtful quality. In the DNCSS study, the sample size was inadequate to meet COSMIN standards.

**Table 4:** Structural validity: rating of measurement properties, study quality, and quality of evidence

			NDRCAQ	DNCS	DNCCS	NCC-PHE	NCASDM	DNPRCS	NPDCCS	CDNMQ
Structural Validity	R		+	?	+	+	+	+	+	+
	QS		D	D	I	VG	VG	VG	VG	VG
	QE		Low	n. a.	Very low	High	High	High	High	High

Note. D = Doubtful; I = Inadequate; n. a. = Not applicable; QE = Quality of Evidence; QS = Quality of Study; R = Rating; VG = Very good; + = Sufficient; ? = Indeterminate

### Reliability and internal consistency: rating of results and quality of evidence

Reliability was assessed in most studies (*n* = 7) using a test–retest procedure. Since the intraclass correlation coefficient (ICC) was ≥ 0.70 in all cases, reliability was rated as sufficient (Table S8-S9 in Additional file 1). These results were supported by an adequate or very good methodological quality of the analyses (see Table 5).

In the DNPRCS study no reliability testing beyond internal consistency was conducted. Therefore, reliability could not be evaluated in these studies.

Internal consistency was assessed in all studies using Cronbach’s alpha (see Additional file 1). As all values exceeded 0.70, internal consistency was rated as sufficient [18] (see Table 5). The quality of implementation was rated as very good in all studies.

**Table 5:** Internal consistency and reliability: rating of measurement properties, study quality, and quality of evidence

		NDRCAQ	DNCS	DNCCS	NCC-PHE	NCASDM	DNPRCS	NPDCCS	CDNMQ
Internal Consistency	R	+	+	+	+	+	+	+	+
	QS	VG	VG	VG	VG	VG	VG	VG	VG
	QE	High	High	High	High	High	High	High	High
Reliability	R	+	+	+	+	+		+	+
	QS	A	A	VG	VG	A		D	D
	QE	Moderate	Moderate	Low	High	Moderate		Low	Very low

Note. I = Adequate; D = Doubtful; QE = Quality of Evidence; QS = Quality of Study; R = Rating; VG = Very good; + = Sufficient

### Cross-cultural validity: rating of results and quality of evidence

Cross-cultural validity is only relevant for the NPDCCS and the CDNMQ. However, it is evaluated exclusively in the CDNMQ (Table 6). Yet, neither multiple group factor analysis nor differential item functioning is conducted, which renders the assessment insufficient. The quality of the study concerning cross-cultural validity is inadequate (Table S10 in Additional file 1).

**Table 6:** Cross-cultural validity: rating of measurement properties, study quality, and quality of evidence

		NDRCAQ	DNCS	DNCCS	NCC-PHE	NCASDM	DNPRCS	NPDCCS	CDNMQ
Cross-cultural Validity/ Measurement Invariance	R								–
	QS								I
	QE								Very low

Note. I = Inadequate; QE = Quality of Evidence; QS = Quality of Study; R = Rating; – = Insufficient

### Construct validity: rating of results and quality of evidence on construct validity

Convergent construct validity was assessed only in the NCC-PHE and DNPRCS study (Table 7). Correlation analyses were conducted for this purpose (Table S11 in Additional file 1). The Nursing Disaster Knowledge Scale was used in the NCC-PHE study, while the Disaster Preparedness Questionnaire was used in the DNPRCS study. As both correlation coefficients were ≥ 0.70, convergent construct validity was rated as sufficient. This rating was supported by the very good methodological quality of the procedures.

**Table 7:** Construct validity: rating of measurement properties, study quality, and quality of evidence

		NDRCAQ	DNCS	DNCCS	NCC-PHE	NCASDM	DNPRCS	NPDCCS	CDNMQ
Construct Validity	R				+		+		
	QS				VG		VG		
	QE				High		High		

Note. QE = Quality of Evidence; QS = Quality of Study; R = Rating; VG = Very good; + = Sufficient

## Discussion

This systematic literature review aimed to identify a self-assessment instrument for measuring disaster nursing competencies as a potential gold standard, to provide a valid and reliable foundation for research, education, and workforce development. In total, eight self-assessment instruments were identified. Although all of them referred to the ICN framework of Disaster Nursing Competencies, they varied substantially in terms of their conceptual foundations and psychometric properties. Overall, based on our assessment, none of the instruments included met the COSMIN criteria for an unqualified recommendation.

The identification of a “best” instrument also proved challenging, partly because many instruments were only partially available. In many cases, they were presented merely in excerpts or in aggregated form. Introductory texts, for instance, detailed item formulations, and explanatory supplementary materials were frequently missing. In addition, inquiries directed to the corresponding authors often remained unanswered. In this context, the term “best instrument” refers to the COSMIN-based recommendation criteria defined in Sect. 2.8, according to which an instrument can only be recommended if all relevant measurement properties are rated as sufficient and supported by high-quality evidence. Among the instruments not fully available, the DNCS appears the most promising, owing to its comprehensively described development process. However, valid conclusions can only be drawn to a limited extent based on the development study alone.

From a methodological perspective, according to the COSMIN standards, which largely correspond to comparable guidelines such as the Standards for Educational and Psychological Testing [17], the NCC-PHE and the CDNMQ perform equally well. Both were the only instruments to receive four sufficient ratings each. For the NCC-PHE, only content validity was rated inconsistently, as the items are available merely in bullet-point form. At the same time, it is the only instrument that developed items for both Level 1 (basic) and Level 2 (advanced) of the Core Competencies, which complicates comparability with other instruments. The CDNMQ received an insufficient rating regarding cross-cultural validity, as neither a multiple group factor analysis nor testing of differential item functioning was conducted. In addition, the instrument itself is not available. When considering content validity separately across all instruments, the DNCS performs best. The quality of evidence, in contrast to the other instruments with sufficient ratings for content validity, is at least at a moderate level. However, this instrument is likewise not available.

In terms of content, the NPCCS appears most promising. Despite its reliance on an earlier version of the ICN framework, it is the only available instrument whose items fully cover all dimensions of this framework. In all other instruments, at least one domain remains unaddressed. At the level of individual competencies, gaps are evident across all instruments, which appears understandable given the total number of 35 competencies. An adequate operationalization with three items per competency would result in a total length of more than 100 items, which calls into question the applicability of such an instrument in everyday nursing or educational practice.

The conceptual inconsistencies identified highlight the need for a clear substantive orientation in the development of future instruments. As an internationally recognized reference for disaster nursing competencies, the ICN framework provides an appropriate standard in this regard. Our analysis, however, shows that while previous instruments cite this framework as their basis, they represent it inconsistently and, in some cases, only partially. Consistent alignment with the ICN framework would, in principle, be desirable, as it could foster comparability and scientific consensus. At the same time, it must be acknowledged that, according to the Standards for Educational and Psychological Testing, a complete operationalization of complex competency frameworks through a single summative test is hardly feasible from a methodological perspective [37]. A pragmatic approach could involve using the ICN framework as an overarching reference, while relying on a manageable reduction of items and, if necessary, the aggregation of subscales. However, it is essential that no domain remains unaddressed and that the conceptual structure of the framework is preserved transparently in order to be able to comprehensively measure and evaluate the extent of defined disaster competencies for nurses. For the scientific community, this implies the task of developing a consensus on the extent to which future instruments should be aligned with the ICN framework and whether a minimal coverage of all domains can be established as a binding standard.

While the substantive and conceptual basis of future instruments is of central importance, their practical applicability must not be overlooked, an aspect commonly summarized in the international literature under the term feasibility [18]. An instrument should be accessible and practical to ensure its feasible use in clinical practice, e.g. regarding nursing training programs or hospital protocols. Particularly if self-assessment instruments are to be used, as emphasized in the introduction, as tools for workforce development, they must be realistically manageable within the working context of nurses. This includes an appropriate completion time, clear and comprehensible item formulations, and a procedure for scoring and interpretation that is as straightforward as possible. Only if these requirements are met can such instruments truly contribute to the advancement of competencies in disaster nursing and be sustainably integrated into research, education, and workforce development.

At the same time, pragmatic requirements for future instruments must be considered. These include their feasibility in everyday nursing practice, appropriate test economy, comprehensibility of items, and straightforward procedures for scoring and interpretation. Equally central is the assurance of accessibility and transparency. Instruments should be freely available, and their development must be documented transparently to enable replicability, international use, and continuous refinement.

### Limitations

This review is based on a comprehensive literature search of seven relevant databases, follows established methodological standards (COSMIN, PRISMA), and includes a double independent appraisal. Nevertheless, several limitations must be acknowledged that restrict the interpretability of the findings. First, it should be noted that the COSMIN checklists were not originally developed for the evaluation of competency measurement instruments but for patient-reported outcome measures. This has direct implications for the assessment of content validity. Whereas COSMIN generally distinguishes between evaluations made by the target group and by experts, no such separation was applied in this review, as these groups frequently overlapped in the included studies.

Furthermore, studies that assessed disaster nursing competencies using non-psychometric approaches were deliberately excluded from the search strategy, in line with the COSMIN standard and the predefined focus of this review on evaluating the measurement properties of self- assessment instruments.

Moreover, for each instrument, only a single study has been conducted, with no additional validation or replication studies published. This lack of multiple assessments limits the robustness of the ratings and the certainty of the conclusions. In contrast to patient-reported outcome measures, for which multiple independent validation studies often exist, and for which content validity is in some cases assessed in separate studies, the evidence base in the field of disaster nursing frequently relies on individual studies. This reduces the possibility of a differentiated assessment of psychometric quality and increases dependence on the quality of the respective study.

In addition, some instruments could not be fully analyzed due to a lack of published items. In such cases, the assessment of content validity had to rely on incomplete or aggregated information, which further complicates comparability across instruments. The quality of the included studies was also frequently limited by insufficient transparency in reporting the development processes.

Finally, this review is restricted to published literature. Therefore, it cannot be excluded that additional relevant instruments exist in the grey literature, which were not captured within the scope of this work.

## Conclusions

This systematic literature review has shown that, to date, no self-assessment instrument for measuring disaster nursing competencies fulfils the methodological and substantive requirements to an extent that would justify an unreserved recommendation or that could be a gold standard. This highlights a clear need for further research: either existing instruments must be systematically refined and improved in terms of both content and psychometrics, or a new instrument must be developed. Regardless of the path chosen, it appears essential to consistently integrate the ICN framework as the conceptual reference to ensure coherent and comparable operationalization of the competence construct. Because the included instruments do not yet meet the methodological quality standards required for evidence-based use, no direct recommendations for nursing training or hospital disaster protocols can be derived from this review. Only if methodological rigor, conceptual basis, and practical applicability are equally addressed can self-assessment instruments realize their potential in research, education, and workforce development in disaster nursing.

## Supplementary Information

Below is the link to the electronic supplementary material.

Supplementary Material 1

Supplementary Material 2

## Data Availability

All data generated or analysed during this study are included in this published article and its supplementary information files.

## Abbreviations

CDNMQ: Competencies for Disaster Nursing Management Questionnaire
COSMIN: Consensus-based Standards for the selection of health Measurement Instruments
DNCS: Disaster Nursing Competency Scale
DNCCS: Disaster Nursing Core Competencies Scale
DNPRCS: Disaster Nursing Preparedness – Response Competency Scale
GRADE: Grading of Recommendations, Assessment, Development and Evaluation
ICC: Intraclass Correlation Coefficient
ICN: International Council of Nurses
INCMCE: International Coalition for Mass Casualty Education
NCASDM: Nurse Competency Assessment Scale in Disaster Management
NCC-PHE: Instrument Nurses‘ Core Competencies for Public Health Emergencies
NDRCAQ: Nurses‘ Disaster Response Competencies Assessment Questionnaire
NPDCCS: Nurses’ Perceptions of Disaster Core Competencies Scale
PRISMA: Preferred Reporting Items for Systematic Reviews and Meta-Analyses
