# Supplementary material for: Measurement properties of self-assessment instruments for disaster nursing competencies: a systematic literature review
Source: BMC Nurs. 2025 Dec 18;25:77. doi: 10.1186/s12912-025-04236-w (PMC12831345; doi:10.1186/s12912-025-04236-w)
Supplement: Supplementary file 1 — Supplementary Material 1 [file 12912_2025_4236_MOESM1_ESM.docx]

**Additional file 1: Procedure of Systematic Literature Search**

**List of Tables**

[**Table S1.** Eligibility Criteria 2](#_Toc207191783)

[**Table S2.** Master Search String 2](#_Toc207191784)

[**Table S3.** Adapted Search Strings 4](#_Toc207191785)

[**Table S4.** Adapted PROMs Search Filter 95](#_Toc207191786)

[**Table S5.** Adapted COSMIN Search Filter for Measurement Properties 95](#_Toc207191787)

[**Table S6.** Summarized Results of Content Validity 96](#_Toc207191788)

[**Table S7.** Summarized Results of Structural Validity 97](#_Toc207191789)

[**Table S8.** Summarized Results of Internal Consistency 97](#_Toc207191790)

[**Table S9.** Summarized Results of Reliability 98](#_Toc207191791)

[**Table S10.** Summarized Results of Cross-cultural Validity 98](#_Toc207191792)

[**Table S11.** Summarized Results of Construct Validity 99](#_Toc207191793)

**Table S1.** Eligibility Criteria

| **Scheme** | **Inclusion criteria** | **Exclusion criteria** |
| --- | --- | --- |
| Construct | Disaster Nursing Competencies | Not all-hazards approach, no competency approach, emergency competencies |
| Population | Nurses | Care assistants, rescue service personnel, and other health care professionals |
| Measurement properties | Any measurement property | Not assessing any measurement properties |
| Type of instrument | Self-assessment scale | External assessment scale, competence diagnostics |

**Table S2.** Master Search String

| **Components** | **Search string** |
| --- | --- |
| Disaster Nursing Competencies | ((disasters[mesh] OR disaster*[tiab] OR "disaster nursing"[mesh]) AND (competenc*[tiab] OR "professional competence"[mesh] OR skill*[tiab] OR knowledge[tiab] OR abilit*[tiab] OR capacit*[tiab] OR capabilit*[tiab] OR judgement*[tiab] OR judgment[mesh] OR attitude*[tiab] OR preparedness[tiab])) |
|  | **AND** |
| Nurses | (nurs*[tiab] OR nurses[mesh]) |
|  | **AND** |
| Instrument properties | (instrumentation[sh] OR methods[sh] OR "Validation Stud*"[pt] OR "Comparative Study"[pt] OR "psychometrics"[MeSH] OR psychometr*[tiab] OR clinimetr*[tw] OR clinometr*[tw] OR "Outcome Assessment, Health Care"[Mesh] OR "outcome assessment"[tiab] OR "outcome measure*"[tw] OR "observer variation"[MeSH] OR "observer variation"[tiab] OR "Health Status Indicators"[Mesh] OR "reproducibility of results"[MeSH] OR reproducib*[tiab] OR "discriminant analysis"[MeSH] OR reliab*[tiab] OR unreliab*[tiab] OR valid*[tiab] OR "coefficient of variation"[tiab] OR coefficient[tiab] OR homogeneity[tiab] OR homogeneous[tiab] OR "internal consistency"[tiab] OR (cronbach*[tiab] AND (alpha[tiab] OR alphas[tiab])) OR (item[tiab] AND (correlation*[tiab] OR selection*[tiab] OR reduction*[tiab])) OR agreement[tw] OR precision[tw] OR imprecision[tw] OR "precise values"[tw] OR test-retest[tiab] OR (test[tiab] AND retest[tiab]) OR (reliab*[tiab] AND (test[tiab] OR retest[tiab])) OR stability[tiab] OR interrater[tiab] OR inter-rater[tiab] OR intrarater[tiab] OR intra-rater[tiab] OR intertester[tiab] OR inter-tester[tiab] OR intratester[tiab] OR intra-tester[tiab] OR interobserver[tiab] OR inter-observer[tiab] OR intraobserver[tiab] OR intra-observer[tiab] OR intertechnician[tiab] OR inter-technician[tiab] OR intratechnician[tiab] OR intra-technician[tiab] OR interexaminer[tiab] OR inter-examiner[tiab] OR intraexaminer[tiab] OR intra-examiner[tiab] OR interassay[tiab] OR inter-assay[tiab] OR intraassay[tiab] OR intra-assay[tiab] OR interindividual[tiab] OR inter-individual[tiab] OR intraindividual[tiab] OR intra-individual[tiab] OR interparticipant[tiab] OR inter-participant[tiab] OR intraparticipant[tiab] OR intra-participant[tiab] OR kappa[tiab] OR kappa’s[tiab] OR kappas[tiab] OR repeatab*[tw] OR ((replicab*[tw] OR repeated[tw]) AND (measure[tw] OR measures[tw] OR findings[tw] OR result[tw] OR results[tw] OR test[tw] OR tests[tw])) OR generaliza*[tiab] OR generalisa*[tiab] OR concordance[tiab] OR (intraclass[tiab] AND correlation*[tiab]) OR discriminative[tiab] OR "known group"[tiab] OR "factor analysis"[tiab] OR "factor analyses"[tiab] OR "factor structure"[tiab] OR "factor structures"[tiab] OR dimension*[tiab] OR subscale*[tiab] OR (multitrait[tiab] AND scaling[tiab] AND (analysis[tiab] OR analyses[tiab])) OR "item discriminant"[tiab] OR "interscale correlation*"[tiab] OR error[tiab] OR errors[tiab] OR "individual variability"[tiab] OR "interval variability"[tiab] OR "rate variability"[tiab] OR (variability[tiab] AND (analysis[tiab] OR values[tiab])) OR (uncertainty[tiab] AND (measurement[tiab] OR measuring[tiab])) OR "standard error of measurement"[tiab] OR sensitiv*[tiab] OR responsive*[tiab] OR (limit[tiab] AND detection[tiab]) OR "minimal detectable concentration"[tiab] OR interpretab*[tiab] OR ((minimal[tiab] OR minimally[tiab] OR clinical[tiab] OR clinically[tiab]) AND (important[tiab] OR significant[tiab] OR detectable[tiab]) AND (change[tiab] OR difference[tiab])) OR (small*[tiab] AND (real[tiab] OR detectable[tiab]) AND (change[tiab] OR difference[tiab])) OR "meaningful change"[tiab] OR "ceiling effect"[tiab] OR "floor effect"[tiab] OR "Item response model"[tiab] OR IRT[tiab] OR Rasch[tiab] OR "Differential item functioning"[tiab] OR DIF[tiab] OR "computer adaptive testing"[tiab] OR "item bank"[tiab] OR "cross-cultural equivalence"[tiab]) |
|  | **AND** |
| Self-assessment scale | (index[tiab] OR indices[tiab] OR instrument[tiab] OR instruments[tiab] OR measure[tiab] OR measures[tiab] OR questionnaire[tiab] OR questionnaires[tiab] OR profile[tiab] OR profiles[tiab] OR scale[tiab] OR scales[tiab] OR score[tiab] OR scores[tiab] OR status[tiab] OR survey[tiab] OR surveys[tiab] OR tool[tiab] OR tools[tiab]) |
|  | **NOT** |
|  | ("address*"[Publication Type] OR "biography"[Publication Type] OR "case reports"[Publication Type] OR "comment"[Publication Type] OR "directory"[Publication Type] OR "editorial"[Publication Type] OR "festschrift"[Publication Type] OR "interview"[Publication Type] OR "lecture*"[Publication Type] OR "legal cas*"[Publication Type] OR "legislation"[Publication Type] OR "letter"[Publication Type] OR "news"[Publication Type] OR "newspaper article"[Publication Type] OR "patient education handout"[Publication Type] OR "popular work*"[Publication Type] OR "congress*"[Publication Type] OR "consensus development conference"[Publication Type] OR "consensus development conference, nih"[Publication Type] OR "practice guideline"[Publication Type]) NOT ("animals"[MeSH Terms] NOT "humans"[MeSH Terms]) |

**Table S3.** Adapted Search Strings

| **Adapted search string (MEDLINE)** | (((competenc*[tiab] OR "professional competence"[mesh] OR skill*[tiab] OR knowledge[tiab] OR abilit*[tiab] OR capacit*[tiab] OR capabilit*[tiab] OR judgement*[tiab] OR judgment[mesh] OR attitude*[tiab] OR preparedness[tiab]) AND (disasters[mesh] OR disaster*[tiab] OR "disaster nursing"[mesh])) AND (nurs*[tiab] OR nurses[mesh]) AND (instrumentation[sh] OR methods[sh] OR "Validation Stud*"[pt] OR "Comparative Study"[pt] OR "psychometrics"[MeSH] OR psychometr*[tiab] OR clinimetr*[tw] OR clinometr*[tw] OR "Outcome Assessment, Health Care"[Mesh] OR "outcome assessment"[tiab] OR "outcome measure*"[tw] OR "observer variation"[MeSH] OR "observer variation"[tiab] OR "Health Status Indicators"[Mesh] OR "reproducibility of results"[MeSH] OR reproducib*[tiab] OR "discriminant analysis"[MeSH] OR reliab*[tiab] OR unreliab*[tiab] OR valid*[tiab] OR "coefficient of variation"[tiab] OR coefficient[tiab] OR homogeneity[tiab] OR homogeneous[tiab] OR "internal consistency"[tiab] OR (cronbach*[tiab] AND (alpha[tiab] OR alphas[tiab])) OR (item[tiab] AND (correlation*[tiab] OR selection*[tiab] OR reduction*[tiab])) OR agreement[tw] OR precision[tw] OR imprecision[tw] OR "precise values"[tw] OR test-retest[tiab] OR (test[tiab] AND retest[tiab]) OR (reliab*[tiab] AND (test[tiab] OR retest[tiab])) OR stability[tiab] OR interrater[tiab] OR inter-rater[tiab] OR intrarater[tiab] OR intra-rater[tiab] OR intertester[tiab] OR inter-tester[tiab] OR intratester[tiab] OR intra-tester[tiab] OR interobserver[tiab] OR inter-observer[tiab] OR intraobserver[tiab] OR intra-observer[tiab] OR intertechnician[tiab] OR inter-technician[tiab] OR intratechnician[tiab] OR intra-technician[tiab] OR interexaminer[tiab] OR inter-examiner[tiab] OR intraexaminer[tiab] OR intra-examiner[tiab] OR interassay[tiab] OR inter-assay[tiab] OR intraassay[tiab] OR intra-assay[tiab] OR interindividual[tiab] OR inter-individual[tiab] OR intraindividual[tiab] OR intra-individual[tiab] OR interparticipant[tiab] OR inter-participant[tiab] OR intraparticipant[tiab] OR intra-participant[tiab] OR kappa[tiab] OR kappa’s[tiab] OR kappas[tiab] OR repeatab*[tw] OR ((replicab*[tw] OR repeated[tw]) AND (measure[tw] OR measures[tw] OR findings[tw] OR result[tw] OR results[tw] OR test[tw] OR tests[tw])) OR generaliza*[tiab] OR generalisa*[tiab] OR concordance[tiab] OR (intraclass[tiab] AND correlation*[tiab]) OR discriminative[tiab] OR "known group"[tiab] OR "factor analysis"[tiab] OR "factor analyses"[tiab] OR "factor structure"[tiab] OR "factor structures"[tiab] OR dimension*[tiab] OR subscale*[tiab] OR (multitrait[tiab] AND scaling[tiab] AND (analysis[tiab] OR analyses[tiab])) OR "item discriminant"[tiab] OR "interscale correlation*"[tiab] OR error[tiab] OR errors[tiab] OR "individual variability"[tiab] OR "interval variability"[tiab] OR "rate variability"[tiab] OR (variability[tiab] AND (analysis[tiab] OR values[tiab])) OR (uncertainty[tiab] AND (measurement[tiab] OR measuring[tiab])) OR "standard error of measurement"[tiab] OR sensitiv*[tiab] OR responsive*[tiab] OR (limit[tiab] AND detection[tiab]) OR "minimal detectable concentration"[tiab] OR interpretab*[tiab] OR ((minimal[tiab] OR minimally[tiab] OR clinical[tiab] OR clinically[tiab]) AND (important[tiab] OR significant[tiab] OR detectable[tiab]) AND (change[tiab] OR difference[tiab])) OR (small*[tiab] AND (real[tiab] OR detectable[tiab]) AND (change[tiab] OR difference[tiab])) OR "meaningful change"[tiab] OR "ceiling effect"[tiab] OR "floor effect"[tiab] OR "Item response model"[tiab] OR IRT[tiab] OR Rasch[tiab] OR "Differential item functioning"[tiab] OR DIF[tiab] OR "computer adaptive testing"[tiab] OR "item bank"[tiab] OR "cross-cultural equivalence"[tiab]) AND (index[tiab] OR indices[tiab] OR instrument[tiab] OR instruments[tiab] OR measure[tiab] OR measures[tiab] OR questionnaire[tiab] OR questionnaires[tiab] OR profile[tiab] OR profiles[tiab] OR scale[tiab] OR scales[tiab] OR score[tiab] OR scores[tiab] OR status[tiab] OR survey[tiab] OR surveys[tiab] OR tool[tiab] OR tools[tiab])) NOT (("address*"[Publication Type] OR "biography"[Publication Type] OR "case reports"[Publication Type] OR "comment"[Publication Type] OR "directory"[Publication Type] OR "editorial"[Publication Type] OR "festschrift"[Publication Type] OR "interview"[Publication Type] OR "lecture*"[Publication Type] OR "legal cas*"[Publication Type] OR "legislation"[Publication Type] OR "letter"[Publication Type] OR "news"[Publication Type] OR "newspaper article"[Publication Type] OR "patient education handout"[Publication Type] OR "popular work*"[Publication Type] OR "congress*"[Publication Type] OR "consensus development conference"[Publication Type] OR "consensus development conference, nih"[Publication Type] OR "practice guideline"[Publication Type]) NOT ("animals"[MeSH Terms] NOT "humans"[MeSH Terms])) |
| --- | --- |
| **Adapted search string (CINAHL)** | \| **#** \| **Query** \| **Limiters/Expanders** \| **Last Run Via** \| **Results** \| \| --- \| --- \| --- \| --- \| --- \| \| S64 \| S63 NOT S62 \| Expanders - Apply equivalent subjects Search modes - Proximity \| Interface - EBSCOhost Research Databases Search Screen - Advanced Search Database - CINAHL \| 9 \| \| S63 \| S17 AND S20 AND S40 AND S41 \| Expanders - Apply equivalent subjects Search modes - Proximity \| Interface - EBSCOhost Research Databases Search Screen - Advanced Search Database - CINAHL \| 9 \| \| S62 \| S42 OR S43 OR S44 OR S45 OR S46 OR S47 OR S48 OR S49 OR S50 OR S51 OR S52 OR S53 OR S54 OR S55 OR S56 OR S57 OR S58 OR S59 OR S60 OR S61 \| Expanders - Apply equivalent subjects Search modes - Proximity \| Interface - EBSCOhost Research Databases Search Screen - Advanced Search Database - CINAHL \| 642,518 \| \| S61 \| PT "practice guideline" \| Expanders - Apply equivalent subjects Search modes - Proximity \| Interface - EBSCOhost Research Databases Search Screen - Advanced Search Database - CINAHL \| 0 \| \| S60 \| PT "consensus development conference, nih" \| Expanders - Apply equivalent subjects Search modes - Proximity \| Interface - EBSCOhost Research Databases Search Screen - Advanced Search Database - CINAHL \| 0 \| \| S59 \| PT "consensus development conference" \| Expanders - Apply equivalent subjects Search modes - Proximity \| Interface - EBSCOhost Research Databases Search Screen - Advanced Search Database - CINAHL \| 0 \| \| S58 \| PT "congress*" \| Expanders - Apply equivalent subjects Search modes - Proximity \| Interface - EBSCOhost Research Databases Search Screen - Advanced Search Database - CINAHL \| 0 \| \| S57 \| PT "popular work*" \| Expanders - Apply equivalent subjects Search modes - Proximity \| Interface - EBSCOhost Research Databases Search Screen - Advanced Search Database - CINAHL \| 0 \| \| S56 \| PT "patient education handout" \| Expanders - Apply equivalent subjects Search modes - Proximity \| Interface - EBSCOhost Research Databases Search Screen - Advanced Search Database - CINAHL \| 0 \| \| S55 \| PT "newspaper article" \| Expanders - Apply equivalent subjects Search modes - Proximity \| Interface - EBSCOhost Research Databases Search Screen - Advanced Search Database - CINAHL \| 0 \| \| S54 \| PT news \| Expanders - Apply equivalent subjects Search modes - Proximity \| Interface - EBSCOhost Research Databases Search Screen - Advanced Search Database - CINAHL \| 0 \| \| S53 \| PT letter \| Expanders - Apply equivalent subjects Search modes - Proximity \| Interface - EBSCOhost Research Databases Search Screen - Advanced Search Database - CINAHL \| 306,203 \| \| S52 \| PT legislation \| Expanders - Apply equivalent subjects Search modes - Proximity \| Interface - EBSCOhost Research Databases Search Screen - Advanced Search Database - CINAHL \| 0 \| \| S51 \| PT "legal cas*" \| Expanders - Apply equivalent subjects Search modes - Proximity \| Interface - EBSCOhost Research Databases Search Screen - Advanced Search Database - CINAHL \| 9,708 \| \| S50 \| PT lecture* \| Expanders - Apply equivalent subjects Search modes - Proximity \| Interface - EBSCOhost Research Databases Search Screen - Advanced Search Database - CINAHL \| 0 \| \| S49 \| PT interview \| Expanders - Apply equivalent subjects Search modes - Proximity \| Interface - EBSCOhost Research Databases Search Screen - Advanced Search Database - CINAHL \| 27,380 \| \| S48 \| PT festschrift \| Expanders - Apply equivalent subjects Search modes - Proximity \| Interface - EBSCOhost Research Databases Search Screen - Advanced Search Database - CINAHL \| 0 \| \| S47 \| PT editorial \| Expanders - Apply equivalent subjects Search modes - Proximity \| Interface - EBSCOhost Research Databases Search Screen - Advanced Search Database - CINAHL \| 285,089 \| \| S46 \| PT directory \| Expanders - Apply equivalent subjects Search modes - Proximity \| Interface - EBSCOhost Research Databases Search Screen - Advanced Search Database - CINAHL \| 0 \| \| S45 \| PT comment \| Expanders - Apply equivalent subjects Search modes - Proximity \| Interface - EBSCOhost Research Databases Search Screen - Advanced Search Database - CINAHL \| 0 \| \| S44 \| PT "case reports" \| Expanders - Apply equivalent subjects Search modes - Proximity \| Interface - EBSCOhost Research Databases Search Screen - Advanced Search Database - CINAHL \| 0 \| \| S43 \| PT biography \| Expanders - Apply equivalent subjects Search modes - Proximity \| Interface - EBSCOhost Research Databases Search Screen - Advanced Search Database - CINAHL \| 15,738 \| \| S42 \| PT address* \| Expanders - Apply equivalent subjects Search modes - Proximity \| Interface - EBSCOhost Research Databases Search Screen - Advanced Search Database - CINAHL \| 0 \| \| S41 \| (MH “Psychometrics”) or ( TI psychometr* or AB psychometr* ) or ( TI clinimetr* or AB clinimetr* ) or ( TI clinometr* OR AB clinometr* ) or (MH “Outcome Assessment”) or ( TI outcome assessment or AB outcome assessment ) or ( TI outcome measure* or AB outcome measure* ) or (MH “Health Status Indicators”) or (MH “Reproducibility of Results”) or (MH “Discriminant Analysis”) or ( ( TI reproducib* or AB reproducib* ) or ( TI reliab* or AB reliab* ) or ( TI unreliab* or AB unreliab* ) ) or ( ( TI valid* or AB valid* ) or ( TI coefficient or AB coefficient ) or ( TI homogeneity or AB homogeneity ) ) or ( TI homogeneous or AB homogeneous ) or ( TI “coefficient of variation” or AB “coefficient of variation” ) or ( TI “internal consistency” or AB “internal consistency” ) or (MH “Internal Consistency+”) or (MH “Reliability+”) or (MH “Measurement Error+”) or (MH “Content Validity+”) or “hypothesis testing” or “structural validity” or “cross-cultural validity” or (MH “Criterion-Related Validity+”) or “responsiveness” or “interpretability” or ( TI reliab* or AB reliab* ) and ( (TI test or AB test) OR (TI retest or AB retest) ) or ( TI stability or AB stability ) or ( TI interrater or AB interrater ) or ( TI inter-rater or AB inter-rater ) or ( TI intrarater or AB intrarater ) or ( TI intra-rater or AB intrarater ) or ( TI intertester or AB intertester) or (TI inter-tester or AB inter-tester) or ( TI intratester or AB intratester) or ( TI intra-tester or AB intra-tester) or ( TI interobserver or AB interobserver) or (TI inter-observer or AB inter-observer ) or ( TI intraobserver or AB intraobserver) or ( TI intra-observer or AB intra-observer) or ( TI intertechnician or AB intertechnician) or (TI inter-technician or AB inter-technician) or ( TI intratechnician or AB intratechnician ) or ( TI intra-technician or AB intra-technician ) or ( TI interexaminer or AB interexaminer ) or (TI inter-examiner or AB inter-examiner) or (TI intraexaminer or AB intraexaminer ) OR (TI intra-examiner or AB intra-examiner ) or (TI intra-examiner or AB intraexaminer ) or (TI interassay or AB interassay ) or ( TI inter-assay or AB inter-assay ) or ( TI intraassay or AB intraassay) or ( TI intra-assay or AB intra-assay ) or (TI interindividual or AB interindividual) or (TI inter-individual or AB inter-individual) OR (TI intraindividual or AB intraindividual) or (TI intra-individual or AB intra-individual) or (TI interparticipant or AB interparticipant) or (TI inter-participant or AB inter-participant ) or (TI intraparticipant or AB intraparticipant) or (TI intra-participant or AB intra-participant ) or (TI kappa or AB kappa) or (TI kappa’s or AB kappa’s ) or (TI kappas or AB kappas) or (TI repeatab* or AB repeatab*) or ( TI responsive* or AB responsive* ) or ( TI interpretab* or AB interpretab* ) \| Expanders - Apply equivalent subjects Search modes - Proximity \| Interface - EBSCOhost Research Databases Search Screen - Advanced Search Database - CINAHL \| 808,180 \| \| S40 \| S21 OR S22 OR S23 OR S24 OR S25 OR S26 OR S27 OR S28 OR S29 OR S30 OR S31 OR S32 OR S33 OR S34 OR S35 OR S36 OR S37 OR S38 OR S39 \| Expanders - Apply equivalent subjects Search modes - Proximity \| Interface - EBSCOhost Research Databases Search Screen - Advanced Search Database - CINAHL \| 1,891,183 \| \| S39 \| XB tools \| Expanders - Apply equivalent subjects Search modes - Proximity \| Interface - EBSCOhost Research Databases Search Screen - Advanced Search Database - CINAHL \| 239,148 \| \| S38 \| XB tool \| Expanders - Apply equivalent subjects Search modes - Proximity \| Interface - EBSCOhost Research Databases Search Screen - Advanced Search Database - CINAHL \| 239,148 \| \| S37 \| XB surveys \| Expanders - Apply equivalent subjects Search modes - Proximity \| Interface - EBSCOhost Research Databases Search Screen - Advanced Search Database - CINAHL \| 342,947 \| \| S36 \| XB survey \| Expanders - Apply equivalent subjects Search modes - Proximity \| Interface - EBSCOhost Research Databases Search Screen - Advanced Search Database - CINAHL \| 342,947 \| \| S35 \| XB status \| Expanders - Apply equivalent subjects Search modes - Proximity \| Interface - EBSCOhost Research Databases Search Screen - Advanced Search Database - CINAHL \| 318,122 \| \| S34 \| XB scores \| Expanders - Apply equivalent subjects Search modes - Proximity \| Interface - EBSCOhost Research Databases Search Screen - Advanced Search Database - CINAHL \| 442,049 \| \| S33 \| XB score \| Expanders - Apply equivalent subjects Search modes - Proximity \| Interface - EBSCOhost Research Databases Search Screen - Advanced Search Database - CINAHL \| 442,049 \| \| S32 \| XB scales \| Expanders - Apply equivalent subjects Search modes - Proximity \| Interface - EBSCOhost Research Databases Search Screen - Advanced Search Database - CINAHL \| 311,917 \| \| S31 \| XB scale \| Expanders - Apply equivalent subjects Search modes - Proximity \| Interface - EBSCOhost Research Databases Search Screen - Advanced Search Database - CINAHL \| 311,917 \| \| S30 \| XB profiles \| Expanders - Apply equivalent subjects Search modes - Proximity \| Interface - EBSCOhost Research Databases Search Screen - Advanced Search Database - CINAHL \| 146,489 \| \| S29 \| XB profile \| Expanders - Apply equivalent subjects Search modes - Proximity \| Interface - EBSCOhost Research Databases Search Screen - Advanced Search Database - CINAHL \| 146,489 \| \| S28 \| XB questionnaires \| Expanders - Apply equivalent subjects Search modes - Proximity \| Interface - EBSCOhost Research Databases Search Screen - Advanced Search Database - CINAHL \| 293,564 \| \| S27 \| XB questionnaire \| Expanders - Apply equivalent subjects Search modes - Proximity \| Interface - EBSCOhost Research Databases Search Screen - Advanced Search Database - CINAHL \| 293,564 \| \| S26 \| XB measures \| Expanders - Apply equivalent subjects Search modes - Proximity \| Interface - EBSCOhost Research Databases Search Screen - Advanced Search Database - CINAHL \| 494,975 \| \| S25 \| XB measure \| Expanders - Apply equivalent subjects Search modes - Proximity \| Interface - EBSCOhost Research Databases Search Screen - Advanced Search Database - CINAHL \| 494,975 \| \| S24 \| XB instruments \| Expanders - Apply equivalent subjects Search modes - Proximity \| Interface - EBSCOhost Research Databases Search Screen - Advanced Search Database - CINAHL \| 85,971 \| \| S23 \| XB instrument \| Expanders - Apply equivalent subjects Search modes - Proximity \| Interface - EBSCOhost Research Databases Search Screen - Advanced Search Database - CINAHL \| 85,971 \| \| S22 \| XB indices \| Expanders - Apply equivalent subjects Search modes - Proximity \| Interface - EBSCOhost Research Databases Search Screen - Advanced Search Database - CINAHL \| 308,216 \| \| S21 \| XB index \| Expanders - Apply equivalent subjects Search modes - Proximity \| Interface - EBSCOhost Research Databases Search Screen - Advanced Search Database - CINAHL \| 308,216 \| \| S20 \| S18 AND S19 \| Expanders - Apply equivalent subjects Search modes - Proximity \| Interface - EBSCOhost Research Databases Search Screen - Advanced Search Database - CINAHL \| 44,987 \| \| S19 \| (MH "Nurses") \| Expanders - Apply equivalent subjects Search modes - Proximity \| Interface - EBSCOhost Research Databases Search Screen - Advanced Search Database - CINAHL \| 71,747 \| \| S18 \| XB nurs* \| Expanders - Apply equivalent subjects Search modes - Proximity \| Interface - EBSCOhost Research Databases Search Screen - Advanced Search Database - CINAHL \| 588,752 \| \| S17 \| S15 AND S16 \| Expanders - Apply equivalent subjects Search modes - Proximity \| Interface - EBSCOhost Research Databases Search Screen - Advanced Search Database - CINAHL \| 5,033 \| \| S16 \| S4 OR S5 OR S6 OR S7 OR S8 OR S9 OR S10 OR S11 OR S12 OR S13 OR S14 \| Expanders - Apply equivalent subjects Search modes - Proximity \| Interface - EBSCOhost Research Databases Search Screen - Advanced Search Database - CINAHL \| 786,358 \| \| S15 \| S1 OR S2 OR S3 \| Expanders - Apply equivalent subjects Search modes - Proximity \| Interface - EBSCOhost Research Databases Search Screen - Advanced Search Database - CINAHL \| 20,374 \| \| S14 \| XB preparedness \| Expanders - Apply equivalent subjects Search modes - Proximity \| Interface - EBSCOhost Research Databases Search Screen - Advanced Search Database - CINAHL \| 12,036 \| \| S13 \| XB attitude* \| Expanders - Apply equivalent subjects Search modes - Proximity \| Interface - EBSCOhost Research Databases Search Screen - Advanced Search Database - CINAHL \| 105,725 \| \| S12 \| (MH "Judgment") \| Expanders - Apply equivalent subjects Search modes - Proximity \| Interface - EBSCOhost Research Databases Search Screen - Advanced Search Database - CINAHL \| 7,688 \| \| S11 \| XB judgement* \| Expanders - Apply equivalent subjects Search modes - Proximity \| Interface - EBSCOhost Research Databases Search Screen - Advanced Search Database - CINAHL \| 7,246 \| \| S10 \| XB capabilit* \| Expanders - Apply equivalent subjects Search modes - Proximity \| Interface - EBSCOhost Research Databases Search Screen - Advanced Search Database - CINAHL \| 25,971 \| \| S9 \| XB capacit* \| Expanders - Apply equivalent subjects Search modes - Proximity \| Interface - EBSCOhost Research Databases Search Screen - Advanced Search Database - CINAHL \| 100,746 \| \| S8 \| XB abilit* \| Expanders - Apply equivalent subjects Search modes - Proximity \| Interface - EBSCOhost Research Databases Search Screen - Advanced Search Database - CINAHL \| 183,599 \| \| S7 \| XB knowledge \| Expanders - Apply equivalent subjects Search modes - Proximity \| Interface - EBSCOhost Research Databases Search Screen - Advanced Search Database - CINAHL \| 289,764 \| \| S6 \| XB skill* \| Expanders - Apply equivalent subjects Search modes - Proximity \| Interface - EBSCOhost Research Databases Search Screen - Advanced Search Database - CINAHL \| 158,124 \| \| S5 \| (MH "Professional Competence") \| Expanders - Apply equivalent subjects Search modes - Proximity \| Interface - EBSCOhost Research Databases Search Screen - Advanced Search Database - CINAHL \| 22,966 \| \| S4 \| XB competenc* \| Expanders - Apply equivalent subjects Search modes - Proximity \| Interface - EBSCOhost Research Databases Search Screen - Advanced Search Database - CINAHL \| 63,401 \| \| S3 \| (MH "Disaster Nursing") \| Expanders - Apply equivalent subjects Search modes - Proximity \| Interface - EBSCOhost Research Databases Search Screen - Advanced Search Database - CINAHL \| 90 \| \| S2 \| XB disaster* \| Expanders - Apply equivalent subjects Search modes - Proximity \| Interface - EBSCOhost Research Databases Search Screen - Advanced Search Database - CINAHL \| 16,097 \| \| S1 \| (MH "Disasters") \| Expanders - Apply equivalent subjects Search modes - Proximity \| Interface - EBSCOhost Research Databases Search Screen - Advanced Search Database - CINAHL \| 7,629 \| |
| **Adapted Search String (Cochrane Library)** | ID Search Hits  #1 MeSH descriptor: [Disasters] explode all trees 2286  #2 MeSH descriptor: [Disaster Nursing] explode all trees 1  #3 (competenc*):ti,ab,kw 14073  #4 MeSH descriptor: [Professional Competence] explode all trees 5531  #5 (skill*):ti,ab,kw 44992  #6 (knowledge):ti,ab,kw 58015  #7 (abilit*):ti,ab,kw 74464  #8 (capacit*):ti,ab,kw 55474  #9 (capabilit*):ti,ab,kw 5900  #10 (judgement*):ti,ab,kw 2783  #11 (attitude*):ti,ab,kw 34544  #12 MeSH descriptor: [Judgment] explode all trees 859  #13 (preparedness):ti,ab,kw 1079  #14 (nurs*):ti,ab,kw 61958  #15 MeSH descriptor: [Nursing] explode all trees 4374  #16 MeSH descriptor: [Methods] this term only 1211  #17 (Validation NEXT Stud*):pt 0  #18 (Comparative NEXT Study):pt 1  #19 MeSH descriptor: [Psychometrics] explode all trees 3950  #20 (clinimetr*) 144  #21 (clinometr*) 12  #22 MeSH descriptor: [Outcome Assessment, Health Care] explode all trees 212377  #23 ("outcome assessment"):ti,ab,kw 96106  #24 (outcome NEXT measure*) 106493  #25 MeSH descriptor: [Observer Variation] explode all trees 2681  #26 MeSH descriptor: [Health Status Indicators] explode all trees 28997  #27 MeSH descriptor: [Reproducibility of Results] explode all trees 16589  #28 (reproducib*):ti,ab,kw 21672  #29 MeSH descriptor: [Discriminant Analysis] explode all trees 229  #30 (reliab*):ti,ab,kw 34785  #31 (unreliab*):ti,ab,kw 1005  #32 (valid*):ti,ab,kw 76112  #33 ("coefficient of variation"):ti,ab,kw 2247  #34 (coefficient):ti,ab,kw 16618  #35 (homogeneity):ti,ab,kw 1865  #36 (homogeneous):ti,ab,kw 3255  #37 ("internal consistency"):ti,ab,kw 2323  #38 (cronbach*):ti,ab,kw 1841  #39 (alpha):ti,ab,kw 49549  #40 (alphas):ti,ab,kw 110  #41 #38 (#39 OR #40) 1549  #42 (item):ti,ab,kw 24981  #43 (correlation*):ti,ab,kw 62208  #44 (selection*):ti,ab,kw 43027  #45 (reduction*):ti,ab,kw 242079  #46 #42 (#43 OR #44 OR #45) 5601  #47 (agreement) 17440  #48 (precision) 11194  #49 (imprecision) 5578  #50 ("precise values") 13  #51 (test-retest):ti,ab,kw 2889  #52 (test):ti,ab,kw 352552  #53 (retest):ti,ab,kw 3465  #54 (#52 AND #53) 3263  #55 (reliab*):ti,ab,kw 34785  #56 (test):ti,ab,kw 352552  #57 (retest):ti,ab,kw 3465  #58 #55 (#56 OR #57) 11862  #59 (stability):ti,ab,kw 23541  #60 (interrater):ti,ab,kw 2948  #61 (inter-rater):ti,ab,kw 1325  #62 (intrarater):ti,ab,kw 847  #63 (intra-rater):ti,ab,kw 365  #64 (intertester):ti,ab,kw 44  #65 (inter-tester):ti,ab,kw 22  #66 (intratester):ti,ab,kw 40  #67 (intra-tester):ti,ab,kw 26  #68 (interobserver):ti,ab,kw 1944  #69 (inter-observer):ti,ab,kw 755  #70 (intraobserver):ti,ab,kw 739  #71 (intra-observer):ti,ab,kw 347  #72 (intertechnician):ti,ab,kw 1  #73 (inter-technician):ti,ab,kw 1  #74 (intratechnician):ti,ab,kw 1  #75 (intra-technician):ti,ab,kw 1  #76 (interexaminer):ti,ab,kw 220  #77 (inter-examiner):ti,ab,kw 137  #78 (intra-examiner):ti,ab,kw 127  #79 (intraexaminer):ti,ab,kw 170  #80 (interassay):ti,ab,kw 146  #81 (inter-assay):ti,ab,kw 103  #82 (intraassay):ti,ab,kw 97  #83 (intra-assay):ti,ab,kw 88  #84 (interindividual):ti,ab,kw 3047  #85 (inter-individual):ti,ab,kw 1374  #86 (intraindividual):ti,ab,kw 2181  #87 (intra-individual):ti,ab,kw 1135  #88 (interparticipant):ti,ab,kw 21  #89 (inter-participant):ti,ab,kw 18  #90 (intraparticipant):ti,ab,kw 28  #91 (intra-participant):ti,ab,kw 19  #92 (kappa):ti,ab,kw 5204  #93 (kappa's):ti,ab,kw 5204  #94 (kappas):ti,ab,kw 54  #95 (repeatab*) 2071  #96 (replicab*) 582  #97 (repeated) 74514  #98 (measure) 110564  #99 (measures) 200397  #100 (findings) 174810  #101 (result) 99180  #102 (results) 1035703  #103 (test) 361551  #104 (tests) 150356  #105 (#96 OR #97) AND (#98 OR #99 OR #100 OR #101 OR #102 OR #103 OR #104) 64457  #106 (generaliza*):ti,ab,kw 5976  #107 (generalisa*):ti,ab,kw 1060  #108 (concordance):ti,ab,kw 4037  #109 (intraclass):ti,ab,kw 3640  #110 (correlation*):ti,ab,kw 62208  #111 #109 AND #110 3559  #112 (discrimiative):ti,ab,kw 0  #113 ("known group"):ti,ab,kw 89  #114 ("factor analysis"):ti,ab,kw 3549  #115 ("factor analyses"):ti,ab,kw 3549  #116 ("factor structure"):ti,ab,kw 520  #117 ("factor structures"):ti,ab,kw 40  #118 (dimension*):ti,ab,kw 30521  #119 (subscale*):ti,ab,kw 16688  #120 (multitrait):ti,ab,kw 48  #121 (scaling):ti,ab,kw 9776  #122 (analysis):ti,ab,kw 587100  #123 (analyses):ti,ab,kw 587100  #124 #120 AND #121 AND (#122 OR #123) 12  #125 ("item discriminant"):ti,ab,kw 5  #126 (interscale NEXT correlation*):ti,ab,kw 5  #127 (error):ti,ab,kw 21281  #128 (errors):ti,ab,kw 10501  #129 ("individual variability"):ti,ab,kw 1214  #130 ("interval variability"):ti,ab,kw 71  #131 ("rate variability"):ti,ab,kw 7671  #132 (variability):ti,ab,kw 29777  #133 (analysis):ti,ab,kw 587100  #134 (values):ti,ab,kw 117489  #135 #132 AND (#133 OR #134) 14716  #136 (uncertainty):ti,ab,kw 7133  #137 (measurement):ti,ab,kw 128824  #138 (measuring):ti,ab,kw 27216  #139 #136 (#137 OR #138) 472  #140 ("standard error of measurement"):ti,ab,kw 201  #141 (sensitiv*):ti,ab,kw 96040  #142 (responsive*):ti,ab,kw 15346  #143 (limit):ti,ab,kw 25554  #144 (detection):ti,ab,kw 35885  #145 #143 AND #144 2167  #146 ("minimal detectable concentration"):ti,ab,kw 1  #147 (interpretab*):ti,ab,kw 767  #148 (minimal):ti,ab,kw 37025  #149 (minimally):ti,ab,kw 13653  #150 (clinical):ti,ab,kw 1072722  #151 (clinically):ti,ab,kw 95514  #152 (important):ti,ab,kw 110625  #153 (significant):ti,ab,kw 578974  #154 (detectable):ti,ab,kw 8974  #155 (change):ti,ab,kw 229695  #156 (difference):ti,ab,kw 317696  #157 (#148 OR #149 OR #150 OR #151) AND (#152 OR #153 OR #154) AND (#155 OR #156) 196619  #158 (small*):ti,ab,kw 126227  #159 (real):ti,ab,kw 35731  #160 (detectable):ti,ab,kw 8974  #161 (change):ti,ab,kw 229695  #162 (difference):ti,ab,kw 317696  #163 #158 AND (#159 OR #160) AND (#161 OR #162) 1563  #164 ("meaningful change"):ti,ab,kw 750  #165 ("ceiling effect"):ti,ab,kw 428  #166 ("floor effect"):ti,ab,kw 128  #167 ("item response model"):ti,ab,kw 7  #168 (irt):ti,ab,kw 409  #169 (rasch):ti,ab,kw 388  #170 ("differential item functioning"):ti,ab,kw 90  #171 (dif):ti,ab,kw 274  #172 ("computer adaptive testing"):ti,ab,kw 21  #173 ("item bank"):ti,ab,kw 65  #174 ("cross-cultural equivalence"):ti,ab,kw 1  #175 (index):ti,ab,kw 232458  #176 (indices):ti,ab,kw 232458  #177 (instrument):ti,ab,kw 15172  #178 (instruments):ti,ab,kw 11930  #179 (measure):ti,ab,kw 103227  #180 (measures):ti,ab,kw 191724  #181 (questionnaire):ti,ab,kw 160932  #182 (questionnaires):ti,ab,kw 85561  #183 (profile):ti,ab,kw 82484  #184 (profiles):ti,ab,kw 28608  #185 (scale):ti,ab,kw 277607  #186 (scales):ti,ab,kw 47910  #187 (score):ti,ab,kw 268329  #188 (scores):ti,ab,kw 185014  #189 (status):ti,ab,kw 174526  #190 (survey):ti,ab,kw 43944  #191 (surveys):ti,ab,kw 50298  #192 (tool):ti,ab,kw 38939  #193 (tools):ti,ab,kw 19502  #194 (disaster*):ti,ab,kw 805  #195 (#1 OR #194 OR #2) AND (#3 OR #4 OR #5 OR #6 OR #7 OR #8 OR #9 OR #10 OR #11 OR #12 OR #13) 737  #196 (#14 OR #15) 62332  #197 #16 OR #17 OR #18 OR #19 OR #20 OR #21 OR #22 OR #23 OR #24 OR #25 OR #26 OR #27 OR #28 OR #29 OR #30 OR #31 OR #32 OR #33 OR #34 OR #35 OR #36 OR #37 OR #41 OR #46 OR #47 OR #48 OR #49 OR #50 OR #51 OR #54 OR #58 OR #59 OR #60 OR #61 OR #62 OR #63 OR #64 OR #65 OR #66 OR #67 OR #68 OR #69 OR #70 OR #71 OR #72 OR #73 OR #74 OR #75 OR #76 OR #77 OR #78 OR #79 OR #80 OR #81 OR #82 OR #83 OR #84 OR #85 OR #86 OR #87 OR #88 OR #89 OR #90 OR #91 OR #92 OR #93 OR #94 OR #95 OR #105 OR #106 OR #107 OR #108 OR #111 OR #112 OR #113 OR #114 OR #115 OR #116 OR #117 OR #118 OR #119 OR #124 OR #125 OR #126 OR #127 OR #128 OR #129 OR #130 OR #131 OR #135 OR #139 OR #140 OR #141 OR #142 OR #145 OR #146 OR #147 OR #157 OR #163 OR #164 OR #165 OR #166 OR #167 OR #168 OR #169 OR #170 OR #171 OR #172 OR #173 OR #174 772478  #198 #175 OR #176 OR #177 OR #178 OR #179 OR #180 OR #181 OR #182 OR #183 OR #184 OR #185 OR #186 OR #187 OR #188 OR #189 OR #190 OR #191 OR #192 OR #193 1018045  #199 (address*):pt 0  #200 (biography):pt 0  #201 ("case reports"):pt 0  #202 (comment):pt 0  #203 (directory):pt 0  #204 (editorial):pt 0  #205 (festschrift):pt 0  #206 (interview):pt 0  #207 (lecture*):pt 0  #208 (legal NEXT cas*):pt 0  #209 (legislation):pt 0  #210 (letter):pt 0  #211 (news):pt 0  #212 ("newspaper article"):pt 0  #213 ("patient education handout"):pt 0  #214 (popular NEXT work*):pt 0  #215 (congress*):pt 0  #216 ("consensus development conference"):pt 0  #217 ("consensus development conference, nih"):pt 0  #218 ("practice guideline"):pt 0  #219 MeSH descriptor: [Animals] explode all trees 889802  #220 MeSH descriptor: [Humans] explode all trees 886521  #221 #199 OR #200 OR #201 OR #202 OR #203 OR #204 OR #205 OR #206 OR #207 OR #208 OR #209 OR #210 OR #211 OR #212 OR #213 OR #214 OR #215 OR #216 OR #217 OR #218 0  #222 #195 AND #196 AND #197 AND #198 64  #223 #222 NOT #221 64 |
| **Adapted Search String (ERIC)** | Search Name: Dissertation Neu  Date Run: 10/04/2025 13:32:23  Comment:  ID Search Hits  #1 MeSH descriptor: [Disasters] explode all trees 2286  #2 MeSH descriptor: [Disaster Nursing] explode all trees 1  #3 (competenc*):ti,ab,kw 14073  #4 MeSH descriptor: [Professional Competence] explode all trees 5531  #5 (skill*):ti,ab,kw 44992  #6 (knowledge):ti,ab,kw 58015  #7 (abilit*):ti,ab,kw 74464  #8 (capacit*):ti,ab,kw 55474  #9 (capabilit*):ti,ab,kw 5900  #10 (judgement*):ti,ab,kw 2783  #11 (attitude*):ti,ab,kw 34544  #12 MeSH descriptor: [Judgment] explode all trees 859  #13 (preparedness):ti,ab,kw 1079  #14 (nurs*):ti,ab,kw 61958  #15 MeSH descriptor: [Nursing] explode all trees 4374  #16 MeSH descriptor: [Methods] this term only 1211  #17 (Validation NEXT Stud*):pt 0  #18 (Comparative NEXT Study):pt 1  #19 MeSH descriptor: [Psychometrics] explode all trees 3950  #20 (clinimetr*) 144  #21 (clinometr*) 12  #22 MeSH descriptor: [Outcome Assessment, Health Care] explode all trees 212377  #23 ("outcome assessment"):ti,ab,kw 96106  #24 (outcome NEXT measure*) 106493  #25 MeSH descriptor: [Observer Variation] explode all trees 2681  #26 MeSH descriptor: [Health Status Indicators] explode all trees 28997  #27 MeSH descriptor: [Reproducibility of Results] explode all trees 16589  #28 (reproducib*):ti,ab,kw 21672  #29 MeSH descriptor: [Discriminant Analysis] explode all trees 229  #30 (reliab*):ti,ab,kw 34785  #31 (unreliab*):ti,ab,kw 1005  #32 (valid*):ti,ab,kw 76112  #33 ("coefficient of variation"):ti,ab,kw 2247  #34 (coefficient):ti,ab,kw 16618  #35 (homogeneity):ti,ab,kw 1865  #36 (homogeneous):ti,ab,kw 3255  #37 ("internal consistency"):ti,ab,kw 2323  #38 (cronbach*):ti,ab,kw 1841  #39 (alpha):ti,ab,kw 49549  #40 (alphas):ti,ab,kw 110  #41 #38 (#39 OR #40) 1549  #42 (item):ti,ab,kw 24981  #43 (correlation*):ti,ab,kw 62208  #44 (selection*):ti,ab,kw 43027  #45 (reduction*):ti,ab,kw 242079  #46 #42 (#43 OR #44 OR #45) 5601  #47 (agreement) 17440  #48 (precision) 11194  #49 (imprecision) 5578  #50 ("precise values") 13  #51 (test-retest):ti,ab,kw 2889  #52 (test):ti,ab,kw 352552  #53 (retest):ti,ab,kw 3465  #54 (#52 AND #53) 3263  #55 (reliab*):ti,ab,kw 34785  #56 (test):ti,ab,kw 352552  #57 (retest):ti,ab,kw 3465  #58 #55 (#56 OR #57) 11862  #59 (stability):ti,ab,kw 23541  #60 (interrater):ti,ab,kw 2948  #61 (inter-rater):ti,ab,kw 1325  #62 (intrarater):ti,ab,kw 847  #63 (intra-rater):ti,ab,kw 365  #64 (intertester):ti,ab,kw 44  #65 (inter-tester):ti,ab,kw 22  #66 (intratester):ti,ab,kw 40  #67 (intra-tester):ti,ab,kw 26  #68 (interobserver):ti,ab,kw 1944  #69 (inter-observer):ti,ab,kw 755  #70 (intraobserver):ti,ab,kw 739  #71 (intra-observer):ti,ab,kw 347  #72 (intertechnician):ti,ab,kw 1  #73 (inter-technician):ti,ab,kw 1  #74 (intratechnician):ti,ab,kw 1  #75 (intra-technician):ti,ab,kw 1  #76 (interexaminer):ti,ab,kw 220  #77 (inter-examiner):ti,ab,kw 137  #78 (intra-examiner):ti,ab,kw 127  #79 (intraexaminer):ti,ab,kw 170  #80 (interassay):ti,ab,kw 146  #81 (inter-assay):ti,ab,kw 103  #82 (intraassay):ti,ab,kw 97  #83 (intra-assay):ti,ab,kw 88  #84 (interindividual):ti,ab,kw 3047  #85 (inter-individual):ti,ab,kw 1374  #86 (intraindividual):ti,ab,kw 2181  #87 (intra-individual):ti,ab,kw 1135  #88 (interparticipant):ti,ab,kw 21  #89 (inter-participant):ti,ab,kw 18  #90 (intraparticipant):ti,ab,kw 28  #91 (intra-participant):ti,ab,kw 19  #92 (kappa):ti,ab,kw 5204  #93 (kappa's):ti,ab,kw 5204  #94 (kappas):ti,ab,kw 54  #95 (repeatab*) 2071  #96 (replicab*) 582  #97 (repeated) 74514  #98 (measure) 110564  #99 (measures) 200397  #100 (findings) 174810  #101 (result) 99180  #102 (results) 1035703  #103 (test) 361551  #104 (tests) 150356  #105 (#96 OR #97) AND (#98 OR #99 OR #100 OR #101 OR #102 OR #103 OR #104) 64457  #106 (generaliza*):ti,ab,kw 5976  #107 (generalisa*):ti,ab,kw 1060  #108 (concordance):ti,ab,kw 4037  #109 (intraclass):ti,ab,kw 3640  #110 (correlation*):ti,ab,kw 62208  #111 #109 AND #110 3559  #112 (discrimiative):ti,ab,kw 0  #113 ("known group"):ti,ab,kw 89  #114 ("factor analysis"):ti,ab,kw 3549  #115 ("factor analyses"):ti,ab,kw 3549  #116 ("factor structure"):ti,ab,kw 520  #117 ("factor structures"):ti,ab,kw 40  #118 (dimension*):ti,ab,kw 30521  #119 (subscale*):ti,ab,kw 16688  #120 (multitrait):ti,ab,kw 48  #121 (scaling):ti,ab,kw 9776  #122 (analysis):ti,ab,kw 587100  #123 (analyses):ti,ab,kw 587100  #124 #120 AND #121 AND (#122 OR #123) 12  #125 ("item discriminant"):ti,ab,kw 5  #126 (interscale NEXT correlation*):ti,ab,kw 5  #127 (error):ti,ab,kw 21281  #128 (errors):ti,ab,kw 10501  #129 ("individual variability"):ti,ab,kw 1214  #130 ("interval variability"):ti,ab,kw 71  #131 ("rate variability"):ti,ab,kw 7671  #132 (variability):ti,ab,kw 29777  #133 (analysis):ti,ab,kw 587100  #134 (values):ti,ab,kw 117489  #135 #132 AND (#133 OR #134) 14716  #136 (uncertainty):ti,ab,kw 7133  #137 (measurement):ti,ab,kw 128824  #138 (measuring):ti,ab,kw 27216  #139 #136 (#137 OR #138) 472  #140 ("standard error of measurement"):ti,ab,kw 201  #141 (sensitiv*):ti,ab,kw 96040  #142 (responsive*):ti,ab,kw 15346  #143 (limit):ti,ab,kw 25554  #144 (detection):ti,ab,kw 35885  #145 #143 AND #144 2167  #146 ("minimal detectable concentration"):ti,ab,kw 1  #147 (interpretab*):ti,ab,kw 767  #148 (minimal):ti,ab,kw 37025  #149 (minimally):ti,ab,kw 13653  #150 (clinical):ti,ab,kw 1072722  #151 (clinically):ti,ab,kw 95514  #152 (important):ti,ab,kw 110625  #153 (significant):ti,ab,kw 578974  #154 (detectable):ti,ab,kw 8974  #155 (change):ti,ab,kw 229695  #156 (difference):ti,ab,kw 317696  #157 (#148 OR #149 OR #150 OR #151) AND (#152 OR #153 OR #154) AND (#155 OR #156) 196619  #158 (small*):ti,ab,kw 126227  #159 (real):ti,ab,kw 35731  #160 (detectable):ti,ab,kw 8974  #161 (change):ti,ab,kw 229695  #162 (difference):ti,ab,kw 317696  #163 #158 AND (#159 OR #160) AND (#161 OR #162) 1563  #164 ("meaningful change"):ti,ab,kw 750  #165 ("ceiling effect"):ti,ab,kw 428  #166 ("floor effect"):ti,ab,kw 128  #167 ("item response model"):ti,ab,kw 7  #168 (irt):ti,ab,kw 409  #169 (rasch):ti,ab,kw 388  #170 ("differential item functioning"):ti,ab,kw 90  #171 (dif):ti,ab,kw 274  #172 ("computer adaptive testing"):ti,ab,kw 21  #173 ("item bank"):ti,ab,kw 65  #174 ("cross-cultural equivalence"):ti,ab,kw 1  #175 (index):ti,ab,kw 232458  #176 (indices):ti,ab,kw 232458  #177 (instrument):ti,ab,kw 15172  #178 (instruments):ti,ab,kw 11930  #179 (measure):ti,ab,kw 103227  #180 (measures):ti,ab,kw 191724  #181 (questionnaire):ti,ab,kw 160932  #182 (questionnaires):ti,ab,kw 85561  #183 (profile):ti,ab,kw 82484  #184 (profiles):ti,ab,kw 28608  #185 (scale):ti,ab,kw 277607  #186 (scales):ti,ab,kw 47910  #187 (score):ti,ab,kw 268329  #188 (scores):ti,ab,kw 185014  #189 (status):ti,ab,kw 174526  #190 (survey):ti,ab,kw 43944  #191 (surveys):ti,ab,kw 50298  #192 (tool):ti,ab,kw 38939  #193 (tools):ti,ab,kw 19502  #194 (disaster*):ti,ab,kw 805  #195 (#1 OR #194 OR #2) AND (#3 OR #4 OR #5 OR #6 OR #7 OR #8 OR #9 OR #10 OR #11 OR #12 OR #13) 737  #196 (#14 OR #15) 62332  #197 #16 OR #17 OR #18 OR #19 OR #20 OR #21 OR #22 OR #23 OR #24 OR #25 OR #26 OR #27 OR #28 OR #29 OR #30 OR #31 OR #32 OR #33 OR #34 OR #35 OR #36 OR #37 OR #41 OR #46 OR #47 OR #48 OR #49 OR #50 OR #51 OR #54 OR #58 OR #59 OR #60 OR #61 OR #62 OR #63 OR #64 OR #65 OR #66 OR #67 OR #68 OR #69 OR #70 OR #71 OR #72 OR #73 OR #74 OR #75 OR #76 OR #77 OR #78 OR #79 OR #80 OR #81 OR #82 OR #83 OR #84 OR #85 OR #86 OR #87 OR #88 OR #89 OR #90 OR #91 OR #92 OR #93 OR #94 OR #95 OR #105 OR #106 OR #107 OR #108 OR #111 OR #112 OR #113 OR #114 OR #115 OR #116 OR #117 OR #118 OR #119 OR #124 OR #125 OR #126 OR #127 OR #128 OR #129 OR #130 OR #131 OR #135 OR #139 OR #140 OR #141 OR #142 OR #145 OR #146 OR #147 OR #157 OR #163 OR #164 OR #165 OR #166 OR #167 OR #168 OR #169 OR #170 OR #171 OR #172 OR #173 OR #174 772478  #198 #175 OR #176 OR #177 OR #178 OR #179 OR #180 OR #181 OR #182 OR #183 OR #184 OR #185 OR #186 OR #187 OR #188 OR #189 OR #190 OR #191 OR #192 OR #193 1018045  #199 (address*):pt 0  #200 (biography):pt 0  #201 ("case reports"):pt 0  #202 (comment):pt 0  #203 (directory):pt 0  #204 (editorial):pt 0  #205 (festschrift):pt 0  #206 (interview):pt 0  #207 (lecture*):pt 0  #208 (legal NEXT cas*):pt 0  #209 (legislation):pt 0  #210 (letter):pt 0  #211 (news):pt 0  #212 ("newspaper article"):pt 0  #213 ("patient education handout"):pt 0  #214 (popular NEXT work*):pt 0  #215 (congress*):pt 0  #216 ("consensus development conference"):pt 0  #217 ("consensus development conference, nih"):pt 0  #218 ("practice guideline"):pt 0  #219 MeSH descriptor: [Animals] explode all trees 889802  #220 MeSH descriptor: [Humans] explode all trees 886521  #221 #199 OR #200 OR #201 OR #202 OR #203 OR #204 OR #205 OR #206 OR #207 OR #208 OR #209 OR #210 OR #211 OR #212 OR #213 OR #214 OR #215 OR #216 OR #217 OR #218 0  #222 #195 AND #196 AND #197 AND #198 64  #223 #222 NOT #221 64 |
| **Adapted Search String (PsychINFO)** | \| **#** \| **Query** \| **Limiters/Expanders** \| **Last Run Via** \| **Results** \| \| --- \| --- \| --- \| --- \| --- \| \| S63 \| S41 NOT S62 \| Expanders - Apply equivalent subjects Search modes - Proximity \| Interface - EBSCOhost Research Databases Search Screen - Advanced Search Database - APA PsycInfo \| 31 \| \| S62 \| S42 OR S43 OR S44 OR S45 OR S46 OR S47 OR S48 OR S49 OR S50 OR S51 OR S52 OR S53 OR S54 OR S55 OR S56 OR S57 OR S58 OR S59 OR S60 OR S61 \| Expanders - Apply equivalent subjects Search modes - Proximity \| Interface - EBSCOhost Research Databases Search Screen - Advanced Search Database - APA PsycInfo \| 0 \| \| S61 \| PT "practice guideline" \| Expanders - Apply equivalent subjects Search modes - Proximity \| Interface - EBSCOhost Research Databases Search Screen - Advanced Search Database - APA PsycInfo \| 0 \| \| S60 \| PT "consensus development conference, nih" \| Expanders - Apply equivalent subjects Search modes - Proximity \| Interface - EBSCOhost Research Databases Search Screen - Advanced Search Database - APA PsycInfo \| 0 \| \| S59 \| PT "consensus development conference" \| Expanders - Apply equivalent subjects Search modes - SmartText Searching \| Interface - EBSCOhost Research Databases Search Screen - Advanced Search Database - APA PsycInfo \| 789 \| \| S58 \| PT congress* \| Expanders - Apply equivalent subjects Search modes - Proximity \| Interface - EBSCOhost Research Databases Search Screen - Advanced Search Database - APA PsycInfo \| 0 \| \| S57 \| PT "popular work*" \| Expanders - Apply equivalent subjects Search modes - Proximity \| Interface - EBSCOhost Research Databases Search Screen - Advanced Search Database - APA PsycInfo \| 0 \| \| S56 \| PT "patient education handout" \| Expanders - Apply equivalent subjects Search modes - Proximity \| Interface - EBSCOhost Research Databases Search Screen - Advanced Search Database - APA PsycInfo \| 0 \| \| S55 \| PT "newspaper article" \| Expanders - Apply equivalent subjects Search modes - Proximity \| Interface - EBSCOhost Research Databases Search Screen - Advanced Search Database - APA PsycInfo \| 0 \| \| S54 \| PT news \| Expanders - Apply equivalent subjects Search modes - Proximity \| Interface - EBSCOhost Research Databases Search Screen - Advanced Search Database - APA PsycInfo \| 0 \| \| S53 \| PT letter \| Expanders - Apply equivalent subjects Search modes - Proximity \| Interface - EBSCOhost Research Databases Search Screen - Advanced Search Database - APA PsycInfo \| 0 \| \| S52 \| PT legislation \| Expanders - Apply equivalent subjects Search modes - Proximity \| Interface - EBSCOhost Research Databases Search Screen - Advanced Search Database - APA PsycInfo \| 0 \| \| S51 \| PT "legal cas*" \| Expanders - Apply equivalent subjects Search modes - Proximity \| Interface - EBSCOhost Research Databases Search Screen - Advanced Search Database - APA PsycInfo \| 0 \| \| S50 \| PT lecture* \| Expanders - Apply equivalent subjects Search modes - Proximity \| Interface - EBSCOhost Research Databases Search Screen - Advanced Search Database - APA PsycInfo \| 0 \| \| S49 \| PT interview \| Expanders - Apply equivalent subjects Search modes - Proximity \| Interface - EBSCOhost Research Databases Search Screen - Advanced Search Database - APA PsycInfo \| 0 \| \| S48 \| PT festschrift \| Expanders - Apply equivalent subjects Search modes - Proximity \| Interface - EBSCOhost Research Databases Search Screen - Advanced Search Database - APA PsycInfo \| 0 \| \| S47 \| PT editorial \| Expanders - Apply equivalent subjects Search modes - Proximity \| Interface - EBSCOhost Research Databases Search Screen - Advanced Search Database - APA PsycInfo \| 0 \| \| S46 \| PT directory \| Expanders - Apply equivalent subjects Search modes - Proximity \| Interface - EBSCOhost Research Databases Search Screen - Advanced Search Database - APA PsycInfo \| 0 \| \| S45 \| PT comment \| Expanders - Apply equivalent subjects Search modes - Proximity \| Interface - EBSCOhost Research Databases Search Screen - Advanced Search Database - APA PsycInfo \| 0 \| \| S44 \| PT "case reports" \| Expanders - Apply equivalent subjects Search modes - Proximity \| Interface - EBSCOhost Research Databases Search Screen - Advanced Search Database - APA PsycInfo \| 0 \| \| S43 \| PT biography \| Expanders - Apply equivalent subjects Search modes - Proximity \| Interface - EBSCOhost Research Databases Search Screen - Advanced Search Database - APA PsycInfo \| 0 \| \| S42 \| PT address* \| Expanders - Apply equivalent subjects Search modes - Proximity \| Interface - EBSCOhost Research Databases Search Screen - Advanced Search Database - APA PsycInfo \| 0 \| \| S41 \| S1 AND S17 AND S20 AND S40 \| Expanders - Apply equivalent subjects Search modes - Proximity \| Interface - EBSCOhost Research Databases Search Screen - Advanced Search Database - APA PsycInfo \| 31 \| \| S40 \| S21 OR S22 OR S23 OR S24 OR S25 OR S26 OR S27 OR S28 OR S29 OR S30 OR S31 OR S32 OR S33 OR S34 OR S35 OR S36 OR S37 OR S38 OR S39 \| Expanders - Apply equivalent subjects Search modes - Proximity \| Interface - EBSCOhost Research Databases Search Screen - Advanced Search Database - APA PsycInfo \| 1,967,213 \| \| S39 \| XB tools \| Expanders - Apply equivalent subjects Search modes - Proximity \| Interface - EBSCOhost Research Databases Search Screen - Advanced Search Database - APA PsycInfo \| 208,101 \| \| S38 \| XB tool \| Expanders - Apply equivalent subjects Search modes - Proximity \| Interface - EBSCOhost Research Databases Search Screen - Advanced Search Database - APA PsycInfo \| 208,101 \| \| S37 \| XB surveys \| Expanders - Apply equivalent subjects Search modes - Proximity \| Interface - EBSCOhost Research Databases Search Screen - Advanced Search Database - APA PsycInfo \| 375,130 \| \| S36 \| XB survey \| Expanders - Apply equivalent subjects Search modes - Proximity \| Interface - EBSCOhost Research Databases Search Screen - Advanced Search Database - APA PsycInfo \| 375,130 \| \| S35 \| XB status \| Expanders - Apply equivalent subjects Search modes - Proximity \| Interface - EBSCOhost Research Databases Search Screen - Advanced Search Database - APA PsycInfo \| 270,346 \| \| S34 \| XB scores \| Expanders - Apply equivalent subjects Search modes - Proximity \| Interface - EBSCOhost Research Databases Search Screen - Advanced Search Database - APA PsycInfo \| 396,177 \| \| S33 \| XB score \| Expanders - Apply equivalent subjects Search modes - Proximity \| Interface - EBSCOhost Research Databases Search Screen - Advanced Search Database - APA PsycInfo \| 396,177 \| \| S32 \| XB scales \| Expanders - Apply equivalent subjects Search modes - Proximity \| Interface - EBSCOhost Research Databases Search Screen - Advanced Search Database - APA PsycInfo \| 460,015 \| \| S31 \| XB scale \| Expanders - Apply equivalent subjects Search modes - Proximity \| Interface - EBSCOhost Research Databases Search Screen - Advanced Search Database - APA PsycInfo \| 460,015 \| \| S30 \| XB profiles \| Expanders - Apply equivalent subjects Search modes - Proximity \| Interface - EBSCOhost Research Databases Search Screen - Advanced Search Database - APA PsycInfo \| 115,591 \| \| S29 \| XB profile \| Expanders - Apply equivalent subjects Search modes - Proximity \| Interface - EBSCOhost Research Databases Search Screen - Advanced Search Database - APA PsycInfo \| 115,591 \| \| S28 \| XB questionnaires \| Expanders - Apply equivalent subjects Search modes - Proximity \| Interface - EBSCOhost Research Databases Search Screen - Advanced Search Database - APA PsycInfo \| 347,978 \| \| S27 \| XB questionnaire \| Expanders - Apply equivalent subjects Search modes - Proximity \| Interface - EBSCOhost Research Databases Search Screen - Advanced Search Database - APA PsycInfo \| 347,978 \| \| S26 \| XB measures \| Expanders - Apply equivalent subjects Search modes - Proximity \| Interface - EBSCOhost Research Databases Search Screen - Advanced Search Database - APA PsycInfo \| 623,165 \| \| S25 \| XB measure \| Expanders - Apply equivalent subjects Search modes - Proximity \| Interface - EBSCOhost Research Databases Search Screen - Advanced Search Database - APA PsycInfo \| 623,165 \| \| S24 \| XB instruments \| Expanders - Apply equivalent subjects Search modes - Proximity \| Interface - EBSCOhost Research Databases Search Screen - Advanced Search Database - APA PsycInfo \| 132,557 \| \| S23 \| XB instrument \| Expanders - Apply equivalent subjects Search modes - Proximity \| Interface - EBSCOhost Research Databases Search Screen - Advanced Search Database - APA PsycInfo \| 132,557 \| \| S22 \| XB indices \| Expanders - Apply equivalent subjects Search modes - Proximity \| Interface - EBSCOhost Research Databases Search Screen - Advanced Search Database - APA PsycInfo \| 168,637 \| \| S21 \| XB index \| Expanders - Apply equivalent subjects Search modes - Proximity \| Interface - EBSCOhost Research Databases Search Screen - Advanced Search Database - APA PsycInfo \| 168,637 \| \| S20 \| S18 OR S19 \| Expanders - Apply equivalent subjects Search modes - Proximity \| Interface - EBSCOhost Research Databases Search Screen - Advanced Search Database - APA PsycInfo \| 125,191 \| \| S19 \| XB nurs* \| Expanders - Apply equivalent subjects Search modes - Proximity \| Interface - EBSCOhost Research Databases Search Screen - Advanced Search Database - APA PsycInfo \| 123,950 \| \| S18 \| DE nurses \| Expanders - Apply equivalent subjects Search modes - Proximity \| Interface - EBSCOhost Research Databases Search Screen - Advanced Search Database - APA PsycInfo \| 38,096 \| \| S17 \| S15 AND S16 \| Expanders - Apply equivalent subjects Search modes - Proximity \| Interface - EBSCOhost Research Databases Search Screen - Advanced Search Database - APA PsycInfo \| 4,359 \| \| S16 \| S4 OR S5 OR S6 OR S7 OR S8 OR S9 OR S10 OR S11 OR S12 OR S13 OR S14 \| Expanders - Apply equivalent subjects Search modes - Proximity \| Interface - EBSCOhost Research Databases Search Screen - Advanced Search Database - APA PsycInfo \| 1,314,211 \| \| S15 \| S2 OR S3 \| Expanders - Apply equivalent subjects Search modes - Proximity \| Interface - EBSCOhost Research Databases Search Screen - Advanced Search Database - APA PsycInfo \| 16,501 \| \| S14 \| XB preparedness \| Expanders - Apply equivalent subjects Search modes - Proximity \| Interface - EBSCOhost Research Databases Search Screen - Advanced Search Database - APA PsycInfo \| 7,627 \| \| S13 \| XB attitude* \| Expanders - Apply equivalent subjects Search modes - Proximity \| Interface - EBSCOhost Research Databases Search Screen - Advanced Search Database - APA PsycInfo \| 244,799 \| \| S12 \| DE judgment \| Expanders - Apply equivalent subjects Search modes - Proximity \| Interface - EBSCOhost Research Databases Search Screen - Advanced Search Database - APA PsycInfo \| 37,328 \| \| S11 \| XB judgement \| Expanders - Apply equivalent subjects Search modes - Proximity \| Interface - EBSCOhost Research Databases Search Screen - Advanced Search Database - APA PsycInfo \| 91,859 \| \| S10 \| XB capabilit* \| Expanders - Apply equivalent subjects Search modes - Proximity \| Interface - EBSCOhost Research Databases Search Screen - Advanced Search Database - APA PsycInfo \| 36,801 \| \| S9 \| XB capacit* \| Expanders - Apply equivalent subjects Search modes - Proximity \| Interface - EBSCOhost Research Databases Search Screen - Advanced Search Database - APA PsycInfo \| 118,978 \| \| S8 \| XB abilit* \| Expanders - Apply equivalent subjects Search modes - Proximity \| Interface - EBSCOhost Research Databases Search Screen - Advanced Search Database - APA PsycInfo \| 348,625 \| \| S7 \| XB knowledge \| Expanders - Apply equivalent subjects Search modes - Proximity \| Interface - EBSCOhost Research Databases Search Screen - Advanced Search Database - APA PsycInfo \| 382,355 \| \| S6 \| XB skill* \| Expanders - Apply equivalent subjects Search modes - Proximity \| Interface - EBSCOhost Research Databases Search Screen - Advanced Search Database - APA PsycInfo \| 281,317 \| \| S5 \| DE "Professional competence" \| Expanders - Apply equivalent subjects Search modes - Proximity \| Interface - EBSCOhost Research Databases Search Screen - Advanced Search Database - APA PsycInfo \| 14,899 \| \| S4 \| XB competenc* \| Expanders - Apply equivalent subjects Search modes - Proximity \| Interface - EBSCOhost Research Databases Search Screen - Advanced Search Database - APA PsycInfo \| 104,266 \| \| S3 \| XB disaster* \| Expanders - Apply equivalent subjects Search modes - Proximity \| Interface - EBSCOhost Research Databases Search Screen - Advanced Search Database - APA PsycInfo \| 15,279 \| \| S2 \| DE Disasters \| Expanders - Apply equivalent subjects Search modes - Proximity \| Interface - EBSCOhost Research Databases Search Screen - Advanced Search Database - APA PsycInfo \| 7,042 \| \| S1 \| (PZ "Validation Stud*" OR PZ "Comparative Study" OR DE "psychometrics" OR XB psychometr* OR TX clinimetr* OR TX clinometr* OR XB "outcome assessment" OR TX "outcome measure*" OR XB "observer variation" OR XB reproducib* OR XB reliab* OR XB unreliab* OR XB valid* OR XB "coefficient of variation" OR XB coefficient OR XB homogeneity OR XB homogeneous OR XB "internal consistency" OR (XB cronbach* AND (XB alpha OR XB alphas)) OR (XB item AND (XB correlation* OR XB selection* OR XB reduction*)) OR TX agreement OR TX precision OR TX imprecision OR TX "precise values" OR XB test-retest OR (XB test AND XB retest) OR (XB reliab* AND (XB test OR XB retest)) OR XB stability OR XB interrater OR XB inter-rater OR XB intrarater OR XB intra-rater OR XB intertester OR XB inter-tester OR XB intratester OR XB intra-tester OR XB interobserver OR XB inter-observer OR XB intraobserver OR XB intra-observer OR XB intertechnician OR XB inter-technician OR XB intratechnician OR XB intra-technician OR XB interexaminer OR XB inter-examiner OR XB intraexaminer OR XB intra-examiner OR XB interassay OR XB inter-assay OR XB intraassay OR XB intra-assay OR XB interindividual OR XB inter-individual OR XB intraindividual OR XB intra-individual OR XB interparticipant OR XB inter-participant OR XB intraparticipant OR XB intra-participant OR XB kappa OR XB kappa’s OR XB kappas OR TX repeatab* OR ((TX replicab* OR TX repeated) AND (TX measure OR TX measures OR TX findings OR TX result OR TX results OR TX test OR TX tests)) OR XB generaliza* OR XB generalisa* OR XB concordance OR (XB intraclass AND XB correlation*) OR XB discriminative OR XB "known group" OR XB "factor analysis" OR XB "factor analyses" OR XB "factor structure" OR XB "factor structures" OR XB dimension* OR XB subscale* OR (XB multitrait AND XB scaling AND (XB analysis OR XB analyses)) OR XB "item discriminant" OR XB "interscale correlation*" OR XB error OR XB errors OR XB "individual variability" OR XB "interval variability" OR XB "rate variability" OR (XB variability AND (XB analysis OR XB values)) OR (XB uncertainty AND (XB measurement OR XB measuring)) OR XB "standard error of measurement" OR XB sensitiv* OR XB responsive* OR (XB limit AND XB detection) OR XB "minimal detectable concentration" OR XB interpretab* OR ((XB minimal OR XB minimally OR XB clinical OR XB clinically) AND (XB important OR XB significant OR XB detectable) AND (XB change OR XB difference)) OR (XB small* AND (XB real OR XB detectable) AND (XB change OR XB difference)) OR XB "meaningful change" OR XB "ceiling effect" OR XB "floor effect" OR XB "Item response model" OR XB IRT OR XB Rasch OR XB "Differential item functioning" OR XB DIF OR XB "computer adaptive testing" OR XB "item bank" OR XB "cross-cultural equivalence") \| Expanders - Apply equivalent subjects Search modes - Proximity \| Interface - EBSCOhost Research Databases Search Screen - Advanced Search Database - APA PsycInfo \| 1,331,987 \| |
| **Adapted Search String (SOCIndex)** | \| **#** \| **Query** \| **Limiters/Expanders** \| **Last Run Via** \| **Results** \| \| --- \| --- \| --- \| --- \| --- \| \| S91 \| S69 NOT S90 \| Expanders - Apply equivalent subjects Search modes - Proximity \| Interface - EBSCOhost Research Databases Search Screen - Advanced Search Database - SocINDEX with Full Text \| 3 \| \| S90 \| S70 OR S71 OR S72 OR S73 OR S74 OR S75 OR S76 OR S77 OR S78 OR S79 OR S80 OR S81 OR S82 OR S83 OR S84 OR S85 OR S86 OR S87 OR S88 OR S89 \| Expanders - Apply equivalent subjects Search modes - Proximity \| Interface - EBSCOhost Research Databases Search Screen - Advanced Search Database - SocINDEX with Full Text \| 18 \| \| S89 \| PT "practice guideline" \| Expanders - Apply equivalent subjects Search modes - SmartText Searching \| Interface - EBSCOhost Research Databases Search Screen - Advanced Search Database - SocINDEX with Full Text \| 564 \| \| S88 \| PT "consensus development conference, nih" \| Expanders - Apply equivalent subjects Search modes - SmartText Searching \| Interface - EBSCOhost Research Databases Search Screen - Advanced Search Database - SocINDEX with Full Text \| 307 \| \| S87 \| PT "consensus development conference" \| Expanders - Apply equivalent subjects Search modes - SmartText Searching \| Interface - EBSCOhost Research Databases Search Screen - Advanced Search Database - SocINDEX with Full Text \| 188 \| \| S86 \| PT congress* \| Expanders - Apply equivalent subjects Search modes - SmartText Searching \| Interface - EBSCOhost Research Databases Search Screen - Advanced Search Database - SocINDEX with Full Text \| 7 \| \| S85 \| PT "popular work*" \| Expanders - Apply equivalent subjects Search modes - SmartText Searching \| Interface - EBSCOhost Research Databases Search Screen - Advanced Search Database - SocINDEX with Full Text \| 156 \| \| S84 \| PT "patient education handout" \| Expanders - Apply equivalent subjects Search modes - SmartText Searching \| Interface - EBSCOhost Research Databases Search Screen - Advanced Search Database - SocINDEX with Full Text \| 21 \| \| S83 \| PT "newspaper article" \| Expanders - Apply equivalent subjects Search modes - SmartText Searching \| Interface - EBSCOhost Research Databases Search Screen - Advanced Search Database - SocINDEX with Full Text \| 198 \| \| S82 \| PT news \| Expanders - Apply equivalent subjects Search modes - SmartText Searching \| Interface - EBSCOhost Research Databases Search Screen - Advanced Search Database - SocINDEX with Full Text \| 5 \| \| S81 \| PT letter \| Expanders - Apply equivalent subjects Search modes - SmartText Searching \| Interface - EBSCOhost Research Databases Search Screen - Advanced Search Database - SocINDEX with Full Text \| 6 \| \| S80 \| PT legislation \| Expanders - Apply equivalent subjects Search modes - SmartText Searching \| Interface - EBSCOhost Research Databases Search Screen - Advanced Search Database - SocINDEX with Full Text \| 9 \| \| S79 \| PT "legal cas*" \| Expanders - Apply equivalent subjects Search modes - SmartText Searching \| Interface - EBSCOhost Research Databases Search Screen - Advanced Search Database - SocINDEX with Full Text \| 496 \| \| S78 \| PT lecture* \| Expanders - Apply equivalent subjects Search modes - SmartText Searching \| Interface - EBSCOhost Research Databases Search Screen - Advanced Search Database - SocINDEX with Full Text \| 1 \| \| S77 \| PT interview \| Expanders - Apply equivalent subjects Search modes - SmartText Searching \| Interface - EBSCOhost Research Databases Search Screen - Advanced Search Database - SocINDEX with Full Text \| 15 \| \| S76 \| PT festschrift \| Expanders - Apply equivalent subjects Search modes - SmartText Searching \| Interface - EBSCOhost Research Databases Search Screen - Advanced Search Database - SocINDEX with Full Text \| 0 \| \| S75 \| PT editorial \| Expanders - Apply equivalent subjects Search modes - SmartText Searching \| Interface - EBSCOhost Research Databases Search Screen - Advanced Search Database - SocINDEX with Full Text \| 2 \| \| S74 \| PT directory \| Expanders - Apply equivalent subjects Search modes - SmartText Searching \| Interface - EBSCOhost Research Databases Search Screen - Advanced Search Database - SocINDEX with Full Text \| 0 \| \| S73 \| PT comment \| Expanders - Apply equivalent subjects Search modes - SmartText Searching \| Interface - EBSCOhost Research Databases Search Screen - Advanced Search Database - SocINDEX with Full Text \| 9 \| \| S72 \| PT "case reports" \| Expanders - Apply equivalent subjects Search modes - SmartText Searching \| Interface - EBSCOhost Research Databases Search Screen - Advanced Search Database - SocINDEX with Full Text \| 134 \| \| S71 \| PT biography \| Expanders - Apply equivalent subjects Search modes - SmartText Searching \| Interface - EBSCOhost Research Databases Search Screen - Advanced Search Database - SocINDEX with Full Text \| 1 \| \| S70 \| PT address* \| Expanders - Apply equivalent subjects Search modes - SmartText Searching \| Interface - EBSCOhost Research Databases Search Screen - Advanced Search Database - SocINDEX with Full Text \| 21 \| \| S69 \| S24 AND S28 AND S67 AND S68 \| Expanders - Apply equivalent subjects Search modes - Proximity \| Interface - EBSCOhost Research Databases Search Screen - Advanced Search Database - SocINDEX with Full Text \| 3 \| \| S68 \| (DE psychometrics OR TI psychometr* OR AB psychometr* OR TX clinimetr* OR TX clinometr* OR TI "outcome assessment" OR AB "outcome assessment" OR TX "outcome measure*" OR TI "observer variation" OR AB "observer variation" OR TI reproducib* OR AB reproducib* OR TI reliab* OR AB reliab* OR TI unreliab* OR AB unreliab* OR TI valid* OR AB valid* OR TI "coefficient of variation" OR AB "coefficient of variation" OR TI coefficient OR AB coefficient OR TI homogeneity OR AB homogeneity OR TI homogeneous OR AB homogeneous OR TI "internal consistency" OR AB "internal consistency" OR (TI cronbach* OR AB cronbach*) AND (TI alpha OR AB alpha OR TI alphas OR AB alphas) OR (TI item OR AB item) AND (TI correlation* OR AB correlation* OR TI selection* OR AB selection* OR TI reduction* OR AB reduction*) OR TX agreement OR TX precision OR TX imprecision OR TX "precise values" OR TI test-retest OR AB test-retest OR (TI test OR AB test) AND (TI retest OR AB retest) OR (TI reliab* OR AB reliab*) AND (TI test OR AB test OR TI retest OR AB retest) OR TI stability OR AB stability OR TI interrater OR AB interrater OR TI inter-rater OR AB inter-rater OR TI intrarater OR AB intrarater OR TI intra-rater OR AB intra-rater OR TI intertester OR AB intertester OR TI inter-tester OR AB inter-tester OR TI intratester OR AB intratester OR TI intra-tester OR AB intra-tester OR TI interobserver OR AB interobserver OR TI inter-observer OR AB inter-observer OR TI intraobserver OR AB intraobserver OR TI intra-observer OR AB intra-observer OR TI intertechnician OR AB intertechnician OR TI inter-technician OR AB inter-technician OR TI intratechnician OR AB intratechnician OR TI intra-technician OR AB intra-technician OR TI interexaminer OR AB interexaminer OR TI inter-examiner OR AB inter-examiner OR TI intraexaminer OR AB intraexaminer OR TI intra-examiner OR AB intra-examiner OR TI interassay OR AB interassay OR TI inter-assay OR AB inter-assay OR TI intraassay OR AB intraassay OR TI intra-assay OR AB intra-assay OR TI interindividual OR AB interindividual OR TI inter-individual OR AB inter-individual OR TI intraindividual OR AB intraindividual OR TI intra-individual OR AB intra-individual OR TI interparticipant OR AB interparticipant OR TI inter-participant OR AB inter-participant OR TI intraparticipant OR AB intraparticipant OR TI intra-participant OR AB intra-participant OR TI kappa OR AB kappa OR TI kappa’s OR AB kappa’s OR TI kappas OR AB kappas TX OR repeatab* OR ((TX replicab* OR TX repeated) AND (TX measure OR TX measures OR TX findings OR TX result OR TX results OR TX test OR TX tests)) OR TI generaliza* OR AB generaliza* OR TI generalisa* OR AB generalisa* OR TI concordance OR AB concordance OR (TI intraclass OR AB intraclass) AND (TI correlation* OR AB correlation*) OR TI discriminative OR AB discriminative OR TI "known group" OR AB "known group" OR TI "factor analysis" OR AB "factor analysis" OR TI "factor analyses" OR AB "factor analyses" OR TI "factor structure" OR AB "factor structure" OR TI "factor structures" OR AB "factor structures" OR TI dimension* OR AB dimension* OR TI subscale* OR AB subscale* OR (TI multitrait OR AB multitrait) AND (TI scaling OR AB scaling) AND (TI analysis OR AB analysis OR TI analyses OR AB analyses) OR TI "item discriminant" OR AB "item discriminant" OR TI "interscale correlation*" OR AB "interscale correlation*" OR TI error OR AB error OR TI errors OR AB errors OR TI "individual variability" OR AB "individual variability" OR TI "interval variability" OR AB "interval variability" OR TI "rate variability" OR AB "rate variability" OR (TI variability OR AB variability) AND (TI analysis OR AB analysis OR TI values OR AB values) OR (TI uncertainty OR AB uncertainty) AND (TI measurement OR AB measurement OR TI measuring OR AB measuring) OR TI "standard error of measurement" OR AB "standard error of measurement" OR TI sensitiv* OR AB sensitiv* OR TI responsive* OR AB responsive* OR (TI limit OR AB limit) AND (TI detection OR AB detection) OR TI "minimal detectable concentration" OR AB "minimal detectable concentration" OR TI interpretab* OR AB interpretab* OR ((TI minimal OR AB minimal OR TI minimally OR AB minimally OR TI clinical OR AB clinical OR TI clinically OR AB clinically) AND (TI important OR AB important OR TI significant OR AB significant OR TI detectable OR AB detectable) AND (TI change OR AB change OR TI difference OR AB difference)) OR (TI small* OR AB small*) AND (TI real OR AB real OR TI detectable OR AB detectable) AND (TI change OR AB change OR TI difference OR AB difference)) OR TI "meaningful change" OR AB "meaningful change" OR TI "ceiling effect" OR AB "ceiling effect" OR TI "floor effect" OR AB "floor effect" OR TI "Item response model" OR AB "Item response model" OR TI IRT OR AB IRT OR TI Rasch OR AB Rasch OR TI "Differential item functioning" OR AB "Differential item functioning" OR TI DIF OR AB DIF OR TI "computer adaptive testing" OR AB "computer adaptive testing" OR TI "item bank" OR AB "item bank" OR TI "cross-cultural equivalence" OR AB "cross-cultural equivalence") \| Expanders - Apply equivalent subjects Search modes - Proximity \| Interface - EBSCOhost Research Databases Search Screen - Advanced Search Database - SocINDEX with Full Text \| 493,828 \| \| S67 \| S29 OR S30 OR S31 OR S32 OR S33 OR S34 OR S35 OR S36 OR S37 OR S38 OR S39 OR S40 OR S41 OR S42 OR S43 OR S44 OR S45 OR S46 OR S47 OR S48 OR S49 OR S50 OR S51 OR S52 OR S53 OR S54 OR S55 OR S56 OR S57 OR S58 OR S59 OR S60 OR S61 OR S62 OR S63 OR S64 OR S65 OR S66 \| Expanders - Apply equivalent subjects Search modes - Proximity \| Interface - EBSCOhost Research Databases Search Screen - Advanced Search Database - SocINDEX with Full Text \| 593,637 \| \| S66 \| AB tools \| Expanders - Apply equivalent subjects Search modes - Proximity \| Interface - EBSCOhost Research Databases Search Screen - Advanced Search Database - SocINDEX with Full Text \| 51,934 \| \| S65 \| TI tools \| Expanders - Apply equivalent subjects Search modes - Proximity \| Interface - EBSCOhost Research Databases Search Screen - Advanced Search Database - SocINDEX with Full Text \| 6,041 \| \| S64 \| AB tool \| Expanders - Apply equivalent subjects Search modes - Proximity \| Interface - EBSCOhost Research Databases Search Screen - Advanced Search Database - SocINDEX with Full Text \| 51,934 \| \| S63 \| TI tool \| Expanders - Apply equivalent subjects Search modes - Proximity \| Interface - EBSCOhost Research Databases Search Screen - Advanced Search Database - SocINDEX with Full Text \| 6,041 \| \| S62 \| AB surveys \| Expanders - Apply equivalent subjects Search modes - Proximity \| Interface - EBSCOhost Research Databases Search Screen - Advanced Search Database - SocINDEX with Full Text \| 158,606 \| \| S61 \| TI surveys \| Expanders - Apply equivalent subjects Search modes - Proximity \| Interface - EBSCOhost Research Databases Search Screen - Advanced Search Database - SocINDEX with Full Text \| 20,208 \| \| S60 \| AB survey \| Expanders - Apply equivalent subjects Search modes - Proximity \| Interface - EBSCOhost Research Databases Search Screen - Advanced Search Database - SocINDEX with Full Text \| 158,606 \| \| S59 \| TI survey \| Expanders - Apply equivalent subjects Search modes - Proximity \| Interface - EBSCOhost Research Databases Search Screen - Advanced Search Database - SocINDEX with Full Text \| 20,208 \| \| S58 \| AB status \| Expanders - Apply equivalent subjects Search modes - Proximity \| Interface - EBSCOhost Research Databases Search Screen - Advanced Search Database - SocINDEX with Full Text \| 115,371 \| \| S57 \| TI status \| Expanders - Apply equivalent subjects Search modes - Proximity \| Interface - EBSCOhost Research Databases Search Screen - Advanced Search Database - SocINDEX with Full Text \| 18,832 \| \| S56 \| AB scores \| Expanders - Apply equivalent subjects Search modes - Proximity \| Interface - EBSCOhost Research Databases Search Screen - Advanced Search Database - SocINDEX with Full Text \| 52,873 \| \| S55 \| TI scores \| Expanders - Apply equivalent subjects Search modes - Proximity \| Interface - EBSCOhost Research Databases Search Screen - Advanced Search Database - SocINDEX with Full Text \| 2,682 \| \| S54 \| AB score \| Expanders - Apply equivalent subjects Search modes - Proximity \| Interface - EBSCOhost Research Databases Search Screen - Advanced Search Database - SocINDEX with Full Text \| 52,873 \| \| S53 \| TI score \| Expanders - Apply equivalent subjects Search modes - Proximity \| Interface - EBSCOhost Research Databases Search Screen - Advanced Search Database - SocINDEX with Full Text \| 2,682 \| \| S52 \| AB scales \| Expanders - Apply equivalent subjects Search modes - Proximity \| Interface - EBSCOhost Research Databases Search Screen - Advanced Search Database - SocINDEX with Full Text \| 88,454 \| \| S51 \| TI scales \| Expanders - Apply equivalent subjects Search modes - Proximity \| Interface - EBSCOhost Research Databases Search Screen - Advanced Search Database - SocINDEX with Full Text \| 14,089 \| \| S50 \| AB scale \| Expanders - Apply equivalent subjects Search modes - Proximity \| Interface - EBSCOhost Research Databases Search Screen - Advanced Search Database - SocINDEX with Full Text \| 88,454 \| \| S49 \| TI scale \| Expanders - Apply equivalent subjects Search modes - Proximity \| Interface - EBSCOhost Research Databases Search Screen - Advanced Search Database - SocINDEX with Full Text \| 14,089 \| \| S48 \| AB profiles \| Expanders - Apply equivalent subjects Search modes - Proximity \| Interface - EBSCOhost Research Databases Search Screen - Advanced Search Database - SocINDEX with Full Text \| 28,978 \| \| S47 \| TI profiles \| Expanders - Apply equivalent subjects Search modes - Proximity \| Interface - EBSCOhost Research Databases Search Screen - Advanced Search Database - SocINDEX with Full Text \| 5,902 \| \| S46 \| AB profile \| Expanders - Apply equivalent subjects Search modes - Proximity \| Interface - EBSCOhost Research Databases Search Screen - Advanced Search Database - SocINDEX with Full Text \| 28,978 \| \| S45 \| TI profile \| Expanders - Apply equivalent subjects Search modes - Proximity \| Interface - EBSCOhost Research Databases Search Screen - Advanced Search Database - SocINDEX with Full Text \| 5,902 \| \| S44 \| AB questionnaires \| Expanders - Apply equivalent subjects Search modes - Proximity \| Interface - EBSCOhost Research Databases Search Screen - Advanced Search Database - SocINDEX with Full Text \| 62,289 \| \| S43 \| TI questionnaires \| Expanders - Apply equivalent subjects Search modes - Proximity \| Interface - EBSCOhost Research Databases Search Screen - Advanced Search Database - SocINDEX with Full Text \| 3,779 \| \| S42 \| AB questionnaire \| Expanders - Apply equivalent subjects Search modes - Proximity \| Interface - EBSCOhost Research Databases Search Screen - Advanced Search Database - SocINDEX with Full Text \| 62,289 \| \| S41 \| TI questionnaire \| Expanders - Apply equivalent subjects Search modes - Proximity \| Interface - EBSCOhost Research Databases Search Screen - Advanced Search Database - SocINDEX with Full Text \| 3,779 \| \| S40 \| AB measures \| Expanders - Apply equivalent subjects Search modes - Proximity \| Interface - EBSCOhost Research Databases Search Screen - Advanced Search Database - SocINDEX with Full Text \| 144,686 \| \| S39 \| TI measures \| Expanders - Apply equivalent subjects Search modes - Proximity \| Interface - EBSCOhost Research Databases Search Screen - Advanced Search Database - SocINDEX with Full Text \| 11,144 \| \| S38 \| AB measure \| Expanders - Apply equivalent subjects Search modes - Proximity \| Interface - EBSCOhost Research Databases Search Screen - Advanced Search Database - SocINDEX with Full Text \| 144,686 \| \| S37 \| TI measure \| Expanders - Apply equivalent subjects Search modes - Proximity \| Interface - EBSCOhost Research Databases Search Screen - Advanced Search Database - SocINDEX with Full Text \| 11,144 \| \| S36 \| AB instruments \| Expanders - Apply equivalent subjects Search modes - Proximity \| Interface - EBSCOhost Research Databases Search Screen - Advanced Search Database - SocINDEX with Full Text \| 32,214 \| \| S35 \| TI instruments \| Expanders - Apply equivalent subjects Search modes - Proximity \| Interface - EBSCOhost Research Databases Search Screen - Advanced Search Database - SocINDEX with Full Text \| 2,982 \| \| S34 \| AB instrument \| Expanders - Apply equivalent subjects Search modes - Proximity \| Interface - EBSCOhost Research Databases Search Screen - Advanced Search Database - SocINDEX with Full Text \| 32,214 \| \| S33 \| TI instrument \| Expanders - Apply equivalent subjects Search modes - Proximity \| Interface - EBSCOhost Research Databases Search Screen - Advanced Search Database - SocINDEX with Full Text \| 2,982 \| \| S32 \| AB indices \| Expanders - Apply equivalent subjects Search modes - Proximity \| Interface - EBSCOhost Research Databases Search Screen - Advanced Search Database - SocINDEX with Full Text \| 39,456 \| \| S31 \| TI indices \| Expanders - Apply equivalent subjects Search modes - Proximity \| Interface - EBSCOhost Research Databases Search Screen - Advanced Search Database - SocINDEX with Full Text \| 8,909 \| \| S30 \| AB index \| Expanders - Apply equivalent subjects Search modes - Proximity \| Interface - EBSCOhost Research Databases Search Screen - Advanced Search Database - SocINDEX with Full Text \| 39,456 \| \| S29 \| TI index \| Expanders - Apply equivalent subjects Search modes - Proximity \| Interface - EBSCOhost Research Databases Search Screen - Advanced Search Database - SocINDEX with Full Text \| 8,909 \| \| S28 \| S25 OR S26 OR S27 \| Expanders - Apply equivalent subjects Search modes - Proximity \| Interface - EBSCOhost Research Databases Search Screen - Advanced Search Database - SocINDEX with Full Text \| 32,305 \| \| S27 \| DE "NURSES" \| Expanders - Apply equivalent subjects Search modes - Proximity \| Interface - EBSCOhost Research Databases Search Screen - Advanced Search Database - SocINDEX with Full Text \| 3,914 \| \| S26 \| AB nurs* \| Expanders - Apply equivalent subjects Search modes - Proximity \| Interface - EBSCOhost Research Databases Search Screen - Advanced Search Database - SocINDEX with Full Text \| 29,184 \| \| S25 \| TI nurs* \| Expanders - Apply equivalent subjects Search modes - Proximity \| Interface - EBSCOhost Research Databases Search Screen - Advanced Search Database - SocINDEX with Full Text \| 13,261 \| \| S24 \| S22 AND S23 \| Expanders - Apply equivalent subjects Search modes - Proximity \| Interface - EBSCOhost Research Databases Search Screen - Advanced Search Database - SocINDEX with Full Text \| 2,216 \| \| S23 \| S3 OR S4 OR S5 OR S6 OR S7 OR S8 OR S9 OR S10 OR S11 OR S12 OR S13 OR S14 OR S15 OR S16 OR S17 OR S18 OR S19 OR S20 OR S21 \| Expanders - Apply equivalent subjects Search modes - Proximity \| Interface - EBSCOhost Research Databases Search Screen - Advanced Search Database - SocINDEX with Full Text \| 381,725 \| \| S22 \| S1 OR S2 \| Expanders - Apply equivalent subjects Search modes - Proximity \| Interface - EBSCOhost Research Databases Search Screen - Advanced Search Database - SocINDEX with Full Text \| 11,393 \| \| S21 \| AB preparedness \| Expanders - Apply equivalent subjects Search modes - Proximity \| Interface - EBSCOhost Research Databases Search Screen - Advanced Search Database - SocINDEX with Full Text \| 2,552 \| \| S20 \| TI preparedness \| Expanders - Apply equivalent subjects Search modes - Proximity \| Interface - EBSCOhost Research Databases Search Screen - Advanced Search Database - SocINDEX with Full Text \| 742 \| \| S19 \| AB attitude* \| Expanders - Apply equivalent subjects Search modes - Proximity \| Interface - EBSCOhost Research Databases Search Screen - Advanced Search Database - SocINDEX with Full Text \| 97,911 \| \| S18 \| TI attitude* \| Expanders - Apply equivalent subjects Search modes - Proximity \| Interface - EBSCOhost Research Databases Search Screen - Advanced Search Database - SocINDEX with Full Text \| 27,698 \| \| S17 \| DE "JUDGMENT (Psychology)" \| Expanders - Apply equivalent subjects Search modes - Proximity \| Interface - EBSCOhost Research Databases Search Screen - Advanced Search Database - SocINDEX with Full Text \| 3,580 \| \| S16 \| AB judgement* \| Expanders - Apply equivalent subjects Search modes - Proximity \| Interface - EBSCOhost Research Databases Search Screen - Advanced Search Database - SocINDEX with Full Text \| 4,256 \| \| S15 \| TI judgement* \| Expanders - Apply equivalent subjects Search modes - Proximity \| Interface - EBSCOhost Research Databases Search Screen - Advanced Search Database - SocINDEX with Full Text \| 818 \| \| S14 \| AB capabilit* \| Expanders - Apply equivalent subjects Search modes - Proximity \| Interface - EBSCOhost Research Databases Search Screen - Advanced Search Database - SocINDEX with Full Text \| 11,761 \| \| S13 \| TI capabilit* \| Expanders - Apply equivalent subjects Search modes - Proximity \| Interface - EBSCOhost Research Databases Search Screen - Advanced Search Database - SocINDEX with Full Text \| 1,452 \| \| S12 \| AB capacit* \| Expanders - Apply equivalent subjects Search modes - Proximity \| Interface - EBSCOhost Research Databases Search Screen - Advanced Search Database - SocINDEX with Full Text \| 41,327 \| \| S11 \| TI capacit* \| Expanders - Apply equivalent subjects Search modes - Proximity \| Interface - EBSCOhost Research Databases Search Screen - Advanced Search Database - SocINDEX with Full Text \| 3,952 \| \| S10 \| AB abilit* \| Expanders - Apply equivalent subjects Search modes - Proximity \| Interface - EBSCOhost Research Databases Search Screen - Advanced Search Database - SocINDEX with Full Text \| 65,824 \| \| S9 \| TI abilit* \| Expanders - Apply equivalent subjects Search modes - Proximity \| Interface - EBSCOhost Research Databases Search Screen - Advanced Search Database - SocINDEX with Full Text \| 4,368 \| \| S8 \| AB knowledge \| Expanders - Apply equivalent subjects Search modes - Proximity \| Interface - EBSCOhost Research Databases Search Screen - Advanced Search Database - SocINDEX with Full Text \| 110,978 \| \| S7 \| TI knowledge \| Expanders - Apply equivalent subjects Search modes - Proximity \| Interface - EBSCOhost Research Databases Search Screen - Advanced Search Database - SocINDEX with Full Text \| 18,388 \| \| S6 \| AB skill* \| Expanders - Apply equivalent subjects Search modes - Proximity \| Interface - EBSCOhost Research Databases Search Screen - Advanced Search Database - SocINDEX with Full Text \| 65,468 \| \| S5 \| TI skill* \| Expanders - Apply equivalent subjects Search modes - Proximity \| Interface - EBSCOhost Research Databases Search Screen - Advanced Search Database - SocINDEX with Full Text \| 10,224 \| \| S4 \| AB competenc* \| Expanders - Apply equivalent subjects Search modes - Proximity \| Interface - EBSCOhost Research Databases Search Screen - Advanced Search Database - SocINDEX with Full Text \| 27,040 \| \| S3 \| TI competenc* \| Expanders - Apply equivalent subjects Search modes - Proximity \| Interface - EBSCOhost Research Databases Search Screen - Advanced Search Database - SocINDEX with Full Text \| 7,066 \| \| S2 \| AB disaster* \| Expanders - Apply equivalent subjects Search modes - Proximity \| Interface - EBSCOhost Research Databases Search Screen - Advanced Search Database - SocINDEX with Full Text \| 10,303 \| \| S1 \| TI disaster* \| Expanders - Apply equivalent subjects Search modes - Proximity \| Interface - EBSCOhost Research Databases Search Screen - Advanced Search Database - SocINDEX with Full Text \| 4,502 \| |
| **Adapted Search String (Embase)** | \| **#** \| **Abfrage** \| **Ergebnisse** \| \| --- \| --- \| --- \| \| 1 \| exp "intermethod comparison"/ \| 313,596 \| \| 2 \| exp "data collection method"/ \| 1,598,562 \| \| 3 \| exp "validation study"/ \| 118,435 \| \| 4 \| exp "feasibility study"/ \| 205,567 \| \| 5 \| exp "pilot study"/ \| 237,013 \| \| 6 \| exp psychometry/ \| 126,574 \| \| 7 \| exp reproducibility/ \| 280,422 \| \| 8 \| "reproducib*".ab,ti. \| 272,283 \| \| 9 \| audit.ab,ti. \| 100,431 \| \| 10 \| "psychometr*".ab,ti. \| 82,094 \| \| 11 \| "clinimetr*".ab,ti. \| 2,246 \| \| 12 \| "clinometr*".ab,ti. \| 87 \| \| 13 \| exp "observer variation"/ \| 21,808 \| \| 14 \| "observer variation".ab,ti. \| 1,955 \| \| 15 \| exp "discriminant analysis"/ \| 32,608 \| \| 16 \| exp validity/ \| 152,525 \| \| 17 \| "reliab*".ab,ti. \| 879,368 \| \| 18 \| "valid*".ab,ti. \| 1,613,742 \| \| 19 \| coefficient.ab,ti. \| 392,131 \| \| 20 \| "internal consistency".ab,ti. \| 50,835 \| \| 21 \| "cronbach*".ab,ti. \| 46,910 \| \| 22 \| alpha.ab,ti. \| 1,343,944 \| \| 23 \| alphas.ab,ti. \| 4,569 \| \| 24 \| 22 or 23 \| 1,346,352 \| \| 25 \| 21 and 24 \| 43,705 \| \| 26 \| "item correlation".ab,ti. \| 675 \| \| 27 \| "item correlations".ab,ti. \| 785 \| \| 28 \| "item selection".ab,ti. \| 978 \| \| 29 \| "item selections".ab,ti. \| 26 \| \| 30 \| "item reduction".ab,ti. \| 1,359 \| \| 31 \| "item reductions".ab,ti. \| 9 \| \| 32 \| agreement.ab,ti. \| 437,659 \| \| 33 \| precision.ab,ti. \| 277,168 \| \| 34 \| imprecision.ab,ti. \| 11,826 \| \| 35 \| "precise values".ab,ti. \| 325 \| \| 36 \| "test-retest".ab,ti. \| 44,288 \| \| 37 \| test.ab,ti. \| 2,965,747 \| \| 38 \| retest.ab,ti. \| 49,223 \| \| 39 \| 37 and 38 \| 46,730 \| \| 40 \| "reliab*".ab,ti. \| 879,368 \| \| 41 \| test.ab,ti. \| 2,965,747 \| \| 42 \| retest.ab,ti. \| 49,223 \| \| 43 \| 41 or 42 \| 2,968,240 \| \| 44 \| 40 and 43 \| 173,345 \| \| 45 \| stability.ab,ti. \| 744,460 \| \| 46 \| interrater.ab,ti. \| 15,053 \| \| 47 \| inter-rater.ab,ti. \| 22,278 \| \| 48 \| intrarater.ab,ti. \| 3,757 \| \| 49 \| intra-rater.ab,ti. \| 5,502 \| \| 50 \| intertester.ab,ti. \| 386 \| \| 51 \| inter-tester.ab,ti. \| 291 \| \| 52 \| intratester.ab,ti. \| 305 \| \| 53 \| intra-tester.ab,ti. \| 223 \| \| 54 \| interobserver.ab,ti. \| 30,212 \| \| 55 \| inter-observer.ab,ti. \| 17,096 \| \| 56 \| intraobserver.ab,ti. \| 10,231 \| \| 57 \| intra-observer.ab,ti. \| 8,123 \| \| 58 \| intertechnician.ab,ti. \| 9 \| \| 59 \| inter-technician.ab,ti. \| 31 \| \| 60 \| intra-technichan.ab,ti. \| 0 \| \| 61 \| intratechnichan.ab,ti. \| 0 \| \| 62 \| interexaminer.ab,ti. \| 980 \| \| 63 \| inter-examiner.ab,ti. \| 1,188 \| \| 64 \| intraexaminer.ab,ti. \| 524 \| \| 65 \| intra-examiner.ab,ti. \| 814 \| \| 66 \| interassay.ab,ti. \| 3,860 \| \| 67 \| inter-assay.ab,ti. \| 7,932 \| \| 68 \| intraassay.ab,ti. \| 1,130 \| \| 69 \| intra-assay.ab,ti. \| 6,254 \| \| 70 \| interindividual.ab,ti. \| 24,647 \| \| 71 \| inter-individual.ab,ti. \| 20,837 \| \| 72 \| intraindividual.ab,ti. \| 8,272 \| \| 73 \| intra-individual.ab,ti. \| 9,071 \| \| 74 \| interparticipant.ab,ti. \| 68 \| \| 75 \| inter-participant.ab,ti. \| 143 \| \| 76 \| intraparticipant.ab,ti. \| 47 \| \| 77 \| intra-participant.ab,ti. \| 92 \| \| 78 \| kappa.ab,ti. \| 151,084 \| \| 79 \| kappas.ab,ti. \| 1,265 \| \| 80 \| "coefficient of variation".ab,ti. \| 39,565 \| \| 81 \| "repeatab*".ab,ti. \| 61,539 \| \| 82 \| "replicab*".ab,ti. \| 8,789 \| \| 83 \| repeated.ab,ti. \| 536,210 \| \| 84 \| measure.ab,ti. \| 1,065,106 \| \| 85 \| measures.ab,ti. \| 1,324,553 \| \| 86 \| findings.ab,ti. \| 3,853,083 \| \| 87 \| result.ab,ti. \| 9,898,057 \| \| 88 \| results.ab,ti. \| 10,812,926 \| \| 89 \| test.ab,ti. \| 2,965,747 \| \| 90 \| tests.ab,ti. \| 1,371,106 \| \| 91 \| 84 or 85 or 86 or 87 or 88 or 89 or 90 \| 21,460,060 \| \| 92 \| 82 or 83 \| 544,795 \| \| 93 \| 91 and 92 \| 397,460 \| \| 94 \| "generaliza*".ab,ti. \| 80,914 \| \| 95 \| "generalisa*".ab,ti. \| 9,936 \| \| 96 \| concordance.ab,ti. \| 103,956 \| \| 97 \| intraclass.ab,ti. \| 48,025 \| \| 98 \| "correlation*".ab,ti. \| 1,953,593 \| \| 99 \| 97 and 98 \| 46,963 \| \| 100 \| discriminative.ab,ti. \| 37,230 \| \| 101 \| "known group".ab,ti. \| 2,143 \| \| 102 \| "factor analysis".ab,ti. \| 72,468 \| \| 103 \| "factor analyses".ab,ti. \| 11,991 \| \| 104 \| "factor structure".ab,ti. \| 21,048 \| \| 105 \| "factor structures".ab,ti. \| 1,653 \| \| 106 \| dimensionality.ab,ti. \| 21,914 \| \| 107 \| "subscale*".ab,ti. \| 87,181 \| \| 108 \| "multitrait scaling analysis".ab,ti. \| 96 \| \| 109 \| "multitrait scaling analyses".ab,ti. \| 23 \| \| 110 \| "item discriminant".ab,ti. \| 144 \| \| 111 \| "interscale correlation".ab,ti. \| 25 \| \| 112 \| "interscale correlations".ab,ti. \| 162 \| \| 113 \| error.ab,ti. \| 381,778 \| \| 114 \| errors.ab,ti. \| 260,501 \| \| 115 \| "measure*".ab,ti. \| 5,706,559 \| \| 116 \| "correlat*".ab,ti. \| 3,368,927 \| \| 117 \| "evaluat*".ab,ti. \| 6,991,149 \| \| 118 \| accuracy.ab,ti. \| 831,982 \| \| 119 \| accurate.ab,ti. \| 688,020 \| \| 120 \| precision.ab,ti. \| 277,168 \| \| 121 \| mean.ab,ti. \| 3,534,135 \| \| 122 \| 115 or 116 or 117 or 118 or 119 or 120 or 121 \| 15,086,184 \| \| 123 \| 113 or 114 \| 578,398 \| \| 124 \| 122 and 123 \| 354,979 \| \| 125 \| "individual variability".ab,ti. \| 16,903 \| \| 126 \| "interval variability".ab,ti. \| 943 \| \| 127 \| "rate variability".ab,ti. \| 36,244 \| \| 128 \| "variability analysis".ab,ti. \| 2,217 \| \| 129 \| uncertainty.ab,ti. \| 143,263 \| \| 130 \| measurement.ab,ti. \| 850,435 \| \| 131 \| measuring.ab,ti. \| 481,799 \| \| 132 \| 130 or 131 \| 1,257,893 \| \| 133 \| 129 and 132 \| 13,174 \| \| 134 \| "standard error of measurement".ab,ti. \| 3,626 \| \| 135 \| "sensitiv*".ab,ti. \| 2,350,993 \| \| 136 \| "responsive*".ab,ti. \| 370,177 \| \| 137 \| limit.ab,ti. \| 473,610 \| \| 138 \| detection.ab,ti. \| 1,546,674 \| \| 139 \| 137 and 138 \| 153,755 \| \| 140 \| "minimal detectable concentration".ab,ti. \| 113 \| \| 141 \| "interpretab*".ab,ti. \| 28,139 \| \| 142 \| "small*".ab,ti. \| 2,824,655 \| \| 143 \| real.ab,ti. \| 946,223 \| \| 144 \| detectable.ab,ti. \| 279,056 \| \| 145 \| change.ab,ti. \| 1,913,791 \| \| 146 \| difference.ab,ti. \| 2,202,188 \| \| 147 \| 145 or 146 \| 3,919,461 \| \| 148 \| 143 or 144 \| 1,216,165 \| \| 149 \| 142 and 147 and 148 \| 17,542 \| \| 150 \| "meaningful change".ab,ti. \| 3,173 \| \| 151 \| "minimal important change".ab,ti. \| 406 \| \| 152 \| "minimal important difference".ab,ti. \| 1,052 \| \| 153 \| "minimally important change".ab,ti. \| 168 \| \| 154 \| "minimally important difference".ab,ti. \| 1,070 \| \| 155 \| "minimal detectable change".ab,ti. \| 2,288 \| \| 156 \| "minimal detectable difference".ab,ti. \| 171 \| \| 157 \| "minimal real change".ab,ti. \| 0 \| \| 158 \| "minimally detectable change".ab,ti. \| 50 \| \| 159 \| "minimally detectable difference".ab,ti. \| 18 \| \| 160 \| "minimal real difference".ab,ti. \| 5 \| \| 161 \| "minimally real change".ab,ti. \| 0 \| \| 162 \| "minimally real difference".ab,ti. \| 0 \| \| 163 \| "ceiling effect".ab,ti. \| 3,592 \| \| 164 \| "floor effect".ab,ti. \| 1,267 \| \| 165 \| "item response model".ab,ti. \| 177 \| \| 166 \| irt.ab,ti. \| 6,465 \| \| 167 \| rasch.ab,ti. \| 7,134 \| \| 168 \| "differential item functioning".ab,ti. \| 2,910 \| \| 169 \| dif.ab,ti. \| 6,762 \| \| 170 \| "computer adaptive testing".ab,ti. \| 399 \| \| 171 \| "item bank".ab,ti. \| 1,213 \| \| 172 \| "cross-cultural equivalence".ab,ti. \| 172 \| \| 173 \| 1 or 2 or 3 or 4 or 5 or 6 or 7 or 8 or 9 or 10 or 11 or 12 or 13 or 14 or 15 or 16 or 17 or 18 or 19 or 20 or 25 or 26 or 27 or 28 or 29 or 30 or 31 or 32 or 33 or 34 or 35 or 36 or 39 or 44 or 45 or 46 or 47 or 48 or 49 or 50 or 51 or 52 or 53 or 54 or 55 or 56 or 57 or 58 or 59 or 60 or 61 or 62 or 63 or 64 or 65 or 66 or 67 or 68 or 69 or 70 or 71 or 72 or 73 or 74 or 75 or 76 or 77 or 78 or 79 or 80 or 81 or 93 or 94 or 95 or 96 or 99 or 100 or 101 or 102 or 103 or 104 or 105 or 106 or 107 or 108 or 109 or 110 or 111 or 112 or 124 or 125 or 126 or 127 or 128 or 133 or 134 or 135 or 136 or 139 or 140 or 141 or 149 or 150 or 151 or 152 or 153 or 154 or 155 or 156 or 157 or 158 or 159 or 160 or 161 or 162 or 163 or 164 or 165 or 166 or 167 or 168 or 169 or 170 or 171 or 172 \| 8,911,080 \| \| 174 \| exp disasters/ \| 39,033 \| \| 175 \| "disaster*".ab,ti. \| 42,143 \| \| 176 \| exp "disaster nursing"/ \| 26 \| \| 177 \| "competenc*".ab,ti. \| 152,748 \| \| 178 \| exp "professional competence"/ \| 35,253 \| \| 179 \| "skill*".ab,ti. \| 382,208 \| \| 180 \| knowledge.ab,ti. \| 1,305,584 \| \| 181 \| "abilit*".ab,ti. \| 1,571,448 \| \| 182 \| "capacit*".ab,ti. \| 1,004,134 \| \| 183 \| "capabilit*".ab,ti. \| 305,326 \| \| 184 \| "judgement*".ab,ti. \| 30,360 \| \| 185 \| exp judgment/ \| 521,946 \| \| 186 \| "attitude*".ab,ti. \| 271,340 \| \| 187 \| preparedness.ab,ti. \| 29,414 \| \| 188 \| 174 or 175 or 176 \| 61,204 \| \| 189 \| 177 or 178 or 179 or 180 or 181 or 182 or 183 or 184 or 185 or 186 or 187 \| 4,872,721 \| \| 190 \| 188 and 189 \| 14,928 \| \| 191 \| "nurs*".ab,ti. \| 692,003 \| \| 192 \| exp nurses/ \| 243,148 \| \| 193 \| 191 or 192 \| 747,318 \| \| 194 \| index.ab,ti. \| 1,666,416 \| \| 195 \| indices.ab,ti. \| 276,855 \| \| 196 \| instrument.ab,ti. \| 207,455 \| \| 197 \| instruments.ab,ti. \| 162,889 \| \| 198 \| measure.ab,ti. \| 1,065,106 \| \| 199 \| measures.ab,ti. \| 1,324,553 \| \| 200 \| questionnaire.ab,ti. \| 864,691 \| \| 201 \| questionnaires.ab,ti. \| 311,635 \| \| 202 \| profile.ab,ti. \| 887,300 \| \| 203 \| profiles.ab,ti. \| 582,998 \| \| 204 \| scale.ab,ti. \| 1,481,401 \| \| 205 \| scales.ab,ti. \| 245,941 \| \| 206 \| score.ab,ti. \| 1,498,314 \| \| 207 \| scores.ab,ti. \| 1,108,616 \| \| 208 \| status.ab,ti. \| 1,743,017 \| \| 209 \| survey.ab,ti. \| 1,031,728 \| \| 210 \| surveys.ab,ti. \| 227,822 \| \| 211 \| tool.ab,ti. \| 1,021,331 \| \| 212 \| tools.ab,ti. \| 549,767 \| \| 213 \| 194 or 195 or 196 or 197 or 198 or 199 or 200 or 201 or 202 or 203 or 204 or 205 or 206 or 207 or 208 or 209 or 210 or 211 or 212 \| 10,454,365 \| \| 214 \| 173 and 190 and 193 and 213 \| 386 \| \| 215 \| "address*".pt. \| 0 \| \| 216 \| biography.pt. \| 0 \| \| 217 \| "case reports".pt. \| 0 \| \| 218 \| comment.pt. \| 0 \| \| 219 \| directory.pt. \| 0 \| \| 220 \| editorial.pt. \| 832,778 \| \| 221 \| festschrift.pt. \| 0 \| \| 222 \| interview.pt. \| 0 \| \| 223 \| "lecture*".pt. \| 0 \| \| 224 \| "legal cas* ".pt. \| 0 \| \| 225 \| legislation.pt. \| 0 \| \| 226 \| letter.pt. \| 1,354,504 \| \| 227 \| news.pt. \| 0 \| \| 228 \| "newspaper article".pt. \| 0 \| \| 229 \| "patient education handout".pt. \| 0 \| \| 230 \| "popular work* ".pt. \| 0 \| \| 231 \| "congress*".pt. \| 0 \| \| 232 \| "consensus development conference".pt. \| 0 \| \| 233 \| "consensus development conference, nih".pt. \| 0 \| \| 234 \| "practice guideline".pt. \| 0 \| \| 235 \| exp animals/ \| 35,753,877 \| \| 236 \| exp humans/ \| 29,098,632 \| \| 237 \| 215 or 216 or 217 or 218 or 219 or 220 or 221 or 222 or 223 or 224 or 225 or 226 or 227 or 228 or 229 or 230 or 231 or 232 or 233 or 234 \| 2,187,282 \| \| 238 \| 214 not 237 \| 386 \| |

**Table S4.** Adapted PROMs Search Filter

| (index[tiab] OR indices[tiab] OR instrument[tiab] OR instruments[tiab] OR measure[tiab] OR measures[tiab] OR questionnaire[tiab] OR questionnaires[tiab] OR profile[tiab] OR profiles[tiab] OR scale[tiab] OR scales[tiab] OR score[tiab] OR scores[tiab] OR status[tiab] OR survey[tiab] OR surveys[tiab] OR tool[tiab] OR tools[tiab]) |
| --- |

**Table S5.** Adapted COSMIN Search Filter for Measurement Properties

| (instrumentation[sh] OR methods[sh] OR "Validation Stud*"[pt] OR "Comparative Study"[pt] OR "psychometrics"[MeSH] OR psychometr*[tiab] OR clinimetr*[tw] OR clinometr*[tw] OR "Outcome Assessment, Health Care"[Mesh] OR "outcome assessment"[tiab] OR "outcome measure*"[tw] OR "observer variation"[MeSH] OR "observer variation"[tiab] OR "Health Status Indicators"[Mesh] OR "reproducibility of results"[MeSH] OR reproducib*[tiab] OR "discriminant analysis"[MeSH] OR reliab*[tiab] OR unreliab*[tiab] OR valid*[tiab] OR "coefficient of variation"[tiab] OR coefficient[tiab] OR homogeneity[tiab] OR homogeneous[tiab] OR "internal consistency"[tiab] OR (cronbach*[tiab] AND (alpha[tiab] OR alphas[tiab])) OR (item[tiab] AND (correlation*[tiab] OR selection*[tiab] OR reduction*[tiab])) OR agreement[tw] OR precision[tw] OR imprecision[tw] OR "precise values"[tw] OR test-retest[tiab] OR (test[tiab] AND retest[tiab]) OR (reliab*[tiab] AND (test[tiab] OR retest[tiab])) OR stability[tiab] OR interrater[tiab] OR inter-rater[tiab] OR intrarater[tiab] OR intra-rater[tiab] OR intertester[tiab] OR inter-tester[tiab] OR intratester[tiab] OR intra-tester[tiab] OR interobserver[tiab] OR inter-observer[tiab] OR intraobserver[tiab] OR intra-observer[tiab] OR intertechnician[tiab] OR inter-technician[tiab] OR intratechnician[tiab] OR intra-technician[tiab] OR interexaminer[tiab] OR inter-examiner[tiab] OR intraexaminer[tiab] OR intra-examiner[tiab] OR interassay[tiab] OR inter-assay[tiab] OR intraassay[tiab] OR intra-assay[tiab] OR interindividual[tiab] OR inter-individual[tiab] OR intraindividual[tiab] OR intra-individual[tiab] OR interparticipant[tiab] OR inter-participant[tiab] OR intraparticipant[tiab] OR intra-participant[tiab] OR kappa[tiab] OR kappa’s[tiab] OR kappas[tiab] OR repeatab*[tw] OR ((replicab*[tw] OR repeated[tw]) AND (measure[tw] OR measures[tw] OR findings[tw] OR result[tw] OR results[tw] OR test[tw] OR tests[tw])) OR generaliza*[tiab] OR generalisa*[tiab] OR concordance[tiab] OR (intraclass[tiab] AND correlation*[tiab]) OR discriminative[tiab] OR "known group"[tiab] OR "factor analysis"[tiab] OR "factor analyses"[tiab] OR "factor structure"[tiab] OR "factor structures"[tiab] OR dimension*[tiab] OR subscale*[tiab] OR (multitrait[tiab] AND scaling[tiab] AND (analysis[tiab] OR analyses[tiab])) OR "item discriminant"[tiab] OR "interscale correlation*"[tiab] OR error[tiab] OR errors[tiab] OR "individual variability"[tiab] OR "interval variability"[tiab] OR "rate variability"[tiab] OR (variability[tiab] AND (analysis[tiab] OR values[tiab])) OR (uncertainty[tiab] AND (measurement[tiab] OR measuring[tiab])) OR "standard error of measurement"[tiab] OR sensitiv*[tiab] OR responsive*[tiab] OR (limit[tiab] AND detection[tiab]) OR "minimal detectable concentration"[tiab] OR interpretab*[tiab] OR ((minimal[tiab] OR minimally[tiab] OR clinical[tiab] OR clinically[tiab]) AND (important[tiab] OR significant[tiab] OR detectable[tiab]) AND (change[tiab] OR difference[tiab])) OR (small*[tiab] AND (real[tiab] OR detectable[tiab]) AND (change[tiab] OR difference[tiab])) OR "meaningful change"[tiab] OR "ceiling effect"[tiab] OR "floor effect"[tiab] OR "Item response model"[tiab] OR IRT[tiab] OR Rasch[tiab] OR "Differential item functioning"[tiab] OR DIF[tiab] OR "computer adaptive testing"[tiab] OR "item bank"[tiab] OR "cross-cultural equivalence"[tiab]) |
| --- |
| **NOT**  ("address*"[Publication Type] OR "biography"[Publication Type] OR "case reports"[Publication Type] OR "comment"[Publication Type] OR "directory"[Publication Type] OR "editorial"[Publication Type] OR "festschrift"[Publication Type] OR "interview"[Publication Type] OR "lecture*"[Publication Type] OR "legal cas*"[Publication Type] OR "legislation"[Publication Type] OR "letter"[Publication Type] OR "news"[Publication Type] OR "newspaper article"[Publication Type] OR "patient education handout"[Publication Type] OR "popular work*"[Publication Type] OR "congress*"[Publication Type] OR "consensus development conference"[Publication Type] OR "consensus development conference, nih"[Publication Type] OR "practice guideline"[Publication Type]) NOT ("animals"[MeSH Terms] NOT "humans"[MeSH Terms]) |

**Table S6.** Summarized Results of Content Validity

| **Instrument** | **Summarized results on content validity** | **Relevance** | |  | **Comprehensiveness** | |  | **Comprehensibility** | |  | **Overall** | |
| --- | --- | --- | --- | --- | --- | --- | --- | --- | --- | --- | --- | --- |
|  |  | **R** | **QS** |  | **R** | **QS** |  | **R** | **QS** |  | **R** | **QE** |
| NDRCAQ | CVI: 0.88 (n = 8) | ± | D |  | – | D |  | ? | D |  | ± | Very low |
| DNCS | CVI: 0.79 (n = 10) | + | D |  | + | D |  | + | D |  | + | Moderate |
| DNCCS | - | ± | D |  | – | D |  | + | D |  | ± | Very low |
| NCC-PHE | CVI: 0.97 (n = 7) | ± | D |  | ± | D |  | + | D |  | ± | Low |
| NCASDM | CVI: 0.95 (n = 15) | ± | D |  | ± | D |  | + | D |  | ± | Moderate |
| DNPRCS | - | ± | A |  | ? | A |  | + | D |  | ± | Low |
| NPDCCS | CVI: 0.93 | ± | A |  | + | D |  | + | D |  | ± | Very low |
| CDNMQ | - | + | D |  | ? | D |  | + | D |  | + | Very low |

*Note.* A = Adequate; D = Doubtful; I = Inadequate; n. a. = Not applicable; QE = Quality of Evidence; QS = Quality of Study; R = Rating; ref = Reference; VG = Very good; + = Sufficient; – = Insufficient; ? = Intermediate; ± = Inconsistent.

**Table S7.** Summarized Results of Structural Validity

| **Instrument** | **Summarized results on structural validity** | **R** | **QS** |
| --- | --- | --- | --- |
| NDRCAQ | EFA: PFA / factor loadings of each item on ist factor ≥ 0.4 | + | D |
| DNCS | EFA | ? | D |
| DNCCS | EFA: PCA explained 72.28 % of the total variance / factor loadings of each item on ist factor ≥ 0.4 | + | I |
| NCC-PHE | EFA and CFA: χ2 (1014) = 4340.173, p < 0.001, with a χ2/df ratio of 2.34. The CFI was 0.907, the TLI was 0.901, the RMSEA was 0.056, and the SRMR was 0.055. | + | VG |
| NCASDM | CFA: χ 2/df = 3.261; NNFI = 0.92; RMSEA = 0.059; GFI = 0.92; RFI = 0.95; NFI = 0.95; CFI = 0.925; IFI = 0.93; RMR = 0.07 | + | VG |
| DNPRCS | CFA: CFI=. 98 , TLI=. 96 , RMSEA= . 10 | + | VG |
| NPDCCS | CFA: χ2 4,006.928 Root mean square error of approximation (RMSEA) 0.061 Standardized root mean square residual (SRMR) 0.0583 The goodness of fit index (GFI) 0.796 Tuckere Lewis index (TLI) 0.907 Incremental fit index (IFI) 0.915 Comparative fit index (CFI) 0.914 | + | VG |
| CDNMQ | EFA/CFA | + | VG |

*Note.* A = Adequate; D = Doubtful; I = Inadequate; n. a. = Not applicable; QS = Quality of Study; R = Rating; ref = Reference; VG = Very good; + = Sufficient; – = Insufficient; ? = Indeterminate; ± = Inconsistent.

**Table S8.** Summarized Results of Internal Consistency

| **Instrument** | **Summarized results on internal consistency** | **R** | **QS** |
| --- | --- | --- | --- |
| NDRCAQ | 0.94 / 0.93 / 0.94 | + | VG |
| DNCS | 0.91 / 0.82 / 0.88 / 0.97 | + | VG |
| DNCCS | 0.98 / 0.92 / 0.86 | + | VG |
| NCC-PHE | 0.966–0.987 | + | VG |
| NCASDM | 0.96 / 0.99 | + | VG |
| DNPRCS | 0.92 / 0.88 / 0.91 / 0.93 | + | VG |
| NPDCCS | 0.884 / 0.916 / 0.942/ 0.945 / 0.941 | + | VG |
| CDNMQ | 0.881 / 0.981 / 0.909 / 0.964 (overall) | + | VG |

*Note.* A = Adequate; D = Doubtful; I = Inadequate; n. a. = Not applicable; QS = Quality of Study; R = Rating; ref = Reference; VG = Very good; + = Sufficient; – = Insufficient; ? = Indeterminate; ± = Inconsistent.

**Table S9.** Summarized Results of Reliability

| **Instrument** | **Summarized results on reliability** | **R** | **QS** |
| --- | --- | --- | --- |
| NDRCAQ | ICC > 0.70 | + | A |
| DNCS | ICC = 0.99 | + | A |
| DNCCS | ICC = 0.76 | + | VG |
| NCC-PHE | ICC = 0.931 | + | VG |
| NCASDM | ICC = 0.98 | + | A |
| DNPRCS |  |  |  |
| NPDCCS | 0.861–0.894 | + | D |
| CDNMQ | > 0.90 | + | D |

*Note.* A = Adequate; D = Doubtful; I = Inadequate; n. a. = Not applicable; QS = Quality of Study; R = Rating; ref = Reference; VG = Very good; + = Sufficient; – = Insufficient; ? = Indeterminate; ± = Inconsistent.

**Table S10.** Summarized Results of Cross-cultural Validity

| **Instrument** | **Summarized results on cross-cultural validity** | **R** | **QS** |
| --- | --- | --- | --- |
| NDRCAQ |  |  |  |
| DNCS |  |  |  |
| DNCCS |  |  |  |
| NCC-PHE |  |  |  |
| NCASDM |  |  |  |
| DNPRCS |  |  |  |
| NPDCCS |  |  |  |
| CDNMQ |  | – | I |

*Note.* A = Adequate; D = Doubtful; I = Inadequate; n. a. = Not applicable; QS = Quality of Study; R = Rating; ref = Reference; VG = Very good; + = Sufficient; – = Insufficient; ? = Indeterminate; ± = Inconsistent.

**Table S11.** Summarized Results of Construct Validity

| **Instrument** | **Summarized results on construct validity** | **R** | **QS** |
| --- | --- | --- | --- |
| NDRCAQ |  |  |  |
| DNCS |  |  |  |
| DNCCS |  |  |  |
| NCC-PHE | r = 0.81 | + | VG |
| NCASDM |  |  |  |
| DNPRCS | r = 0.7 / r = 0.71 / r = 0.78 | + | VG |
| NPDCCS |  |  |  |
| CDNMQ |  |  |  |

*Note.* A = Adequate; D = Doubtful; I = Inadequate; n. a. = Not applicable; QS = Quality of Study; R = Rating; ref = Reference; VG = Very good; + = Sufficient; – = Insufficient; ? = Indeterminate; ± = Inconsistent.
